# An updated assessment of the molecular prevalence and risk factors of *Babesia* infection among crossbred cattle: a diagnostic cross-sectional study

**DOI:** 10.1186/s12917-026-05639-w

**Published:** 2026-06-26

**Authors:** Ahmed Gareh, Hesham A. Sadek, Ahmed Kamal Dyab, Mohsen I. Arafa, Gehan Mohamed Sayed, Basem Refat Nageib

**Affiliations:** 1 Department of Parasitology, Faculty of Veterinary Medicine, Aswan University, 81528 Aswan, Egypt; 2 Parasitology Department, Assiut Lab, Agriculture Research Center, Animal Health Research Institute (AHRI), Assiut, Egypt; 3 Department of Medical Parasitology, Faculty of Medicine, Assiut University, Assiut, 71526 Egypt; 4 Department of Parasitology, School of Veterinary Medicine, Badr University in Assiut, New Nasser City, Assiut, Egypt

**Keywords:** *Babesia bovis*, *Babesia bigemina*, Cattle, Egypt, PCR, Ticks

## Abstract

**Background:**

Bovine babesiosis is a hemoprotozoal disease that poses a serious danger to cattle productivity in tropical and subtropical areas. The goal of the current study is to update the data regarding *Babesia* spp. prevalence. Assessing the risk variables linked to the illness in crossbred cattle from January to December 2025 in Assiut, Egypt.

**Methods:**

A total of 200 blood samples were obtained from crossbred cattle, including 84 apparently healthy and 116 showing clinical signs suggestive of babesiosis. Giemsa-stained blood smears were inspected microscopically, and molecular analysis that targeted the *Babesia* 18 S rRNA gene verified the results.

**Results:**

The overall prevalence of *Babesia* spp. infection detected microscopically was 6% (12/200). The percentage of animals with symptoms was 8.6% higher than in apparently healthy ones. Epidemiological analysis showed higher infection rates in males (8.33%) compared to females (4.3%), and in cattle younger than one year (8.75%). There was a discernible seasonal variation in infestation rates, which peaked at 7.7% during the hot season and dropped to 4.2% over the cold season, even though age, sex, and season were not statistically significant (*P* > 0.05). A significant association was found between *Babesia* infection and hard tick infestation (OR = 21.64; 95% CI: 2.35–199.12; *P* = 0.001) suggesting that ticks play a critical role in the spread of disease. The presence of *B. bovis* and *B. bigemina* in crossbred cattle in Assiut Province was confirmed by molecular confirmation of a few heavily infected samples using PCR targeting the *Babesia* 18 S rRNA gene.

**Conclusions:**

For the first time, this work uses combination microscopy and PCR to confirm the existence of two *Babesia* species (*B. bovis* and *B. bigemina*) in Assiut province, upper Egypt. The data indicate that the parasite is actively circulating, emphasizing the significance of good tick management and increased diagnostic surveillance in order to prevent economic losses and maintain animal health.

**Supplementary Information:**

The online version contains supplementary material available at 10.1186/s12917-026-05639-w.

## Introduction

Bovine babesiosis is a ruminant parasite illness caused by intra-erythrocytic *Babesia* spp. that is transmitted by hard ticks. The genus *Babesia* belongs to the phylum Apicomplexa, class Sporozoasida, order Eucoccidiorida, suborder Piroplasmorina, and family Babesiidae [1, 2].

Babesiosis is responsible for financial losses due to decreased milk and meat production, mortality, and management and control trial claims [3]. Additionally, the zoonotic potential of the *Babesia* parasite led to curiosity in human medicine [4]. Approximately 250 different *Babesia* species have been identified globally, with 48 officially recognized in taxonomy and roughly 10 known to infect humans [5].

The three most recognized and virulent *Babesia* species that infect cattle globally are *B. bovis*,* B. bigemina*, and *B. divergens*. Conversely, *B. major*,* B. ovate*, and *B. occultans* exhibit low pathogenicity [6]. In Egypt, the two predominant pathogenic species affecting cattle are *B. bigemina* and *B. bovis* [7].

Hard ticks, especially *Rhipicephalus* sp., the main *Babesia* vector, have been shown to transmit bovine babesiosis. Other tick species that have been discovered as vectors of babesiosis include *Boophilus decoloratus*, *Hyalomma* sp., and *Ixodes ricinus* [2]. The infected hard tick (final host) injects infectious sporozoites into the cow intermediate host [8].

The most typical signs of acute babesiosis are fever, hemolytic anemia, icterus, inappetence, and hemoglobinuria, whereas chronic infections are usually asymptomatic [9]. Long-term illness has been linked to pale to icteric mucous membranes, respiratory troubles, and general weakness [10, 11]. The fever during diseases can cause abortions in pregnant cattle. The severity of the disease is influenced by the number of infecting microorganisms, age, parasite strain, and bovine immune state [12].

To identify blood parasites, thick and thin blood smears stained with Giemsa are often inspected under a microscope. This approach is often used to diagnose acute babesiosis. It is less expensive and requires less time than most other methods [13]. However, this approach is rarely successful in identifying chronic babesiosis due to the low parasitemia. Furthermore, recognizing different species of *Babesia* is the main challenge in using this method [14].

As a result, developing a reliable and accurate diagnostic technique is critical for determining the true severity of *Babesia* infection. While microscopic inspection of Giemsa-stained blood smears remains the traditional and practical gold standard for mass screening, Polymerase Chain Reaction (PCR) is a powerful, extremely sensitive confirmation method. Using molecular amplification to validate microscopically observed instances ensures unequivocal species confirmation while eliminating potential observer bias [15, 16].

To the best of the authors’ knowledge, the Assiut Governorate in Upper Egypt has limited data about molecular research on bovine babesiosis. Consequently, we clearly stated that the current study was designed to provide a more accurate and comprehensive assessment of *Babesia* prevalence in Assiut province, Upper Egypt by combining microscopic and molecular approaches, as well as assessing associated risk factors and the role of tick infestation in disease transmission.

## Materials and methods

### Study design

A descriptive cross-sectional study was conducted to investigate the prevalence and molecular characteristics of *Babesia* infection in animals. To maintain an organized epidemiological design in the study, the key design factors, such as timeframe, geographical location, and baseline animal characteristics, are described systematically in the following subsections.

### Study area

Between January and December of 2025, this study was carried out in the Assiut Governorate, Egypt, which is roughly positioned at latitude 27^0^11’00” N and longitude 31^0^10’00” E. Assiut has a desert climate with an average yearly temperature of 23 °C (1 °C to 45 °C) and little precipitation. According to [17], the region’s humidity and other climatic characteristics provide the ideal conditions for the flurry of blood parasites. The geographic coordinates of the sampling sites were acquired with a handheld Global Positioning System (GPS) receiver to assure spatial accuracy. As this is a cross-sectional study, sample collection and animal data recording were performed concurrently at a single visit, with no follow-up period necessary.

### Animals data

To assess the study’s eligibility criteria 200 crossbred cattle randomly selected from various private farms in Assiut Governorate. The inclusion criteria required that animals demonstrate particular clinical indications of vector-borne diseases, such as pyrexia, hemoglobinuria, or active tick infestation. From the randomly surveyed population, 116 clinically affected animals met these standards and were enrolled in the study. Animals receiving active acaricidal or antiprotozoal medication or if they presented with confounding systemic diseases unrelated to tick-borne infections were excluded. The animals were divided into three age groups: 80 calves (less than a year old), 56 young cattle (one to three years old), and 64 adults (beyond three years old). The sample set consisted of 116 females and 84 males. The samples were collected in both the hot and cold seasons. It is noteworthy that in Upper Egypt, the cold season (December–February) is defined by temperatures between 15 and 27 °C, while the hot season (March–November) is defined by temperatures above 28 °C [18].

### Animal population and investigated variable

To assess the epidemiology of *Babesia* infection in the study area, the key outcome variable was the presence or absence of blood parasite infection (confirmed by microscopy or PCR). The independent variables were host-related (clinical status, age groups, gender, season, and tick infestation). To adjust for potential confounders, strong exclusion criteria were used: animals with concurrent systemic or infectious illnesses (e.g., severe bacterial mastitis or respiratory complexes) that could produce pyrexia or hematuria were eliminated. Furthermore, only crossbred cattle under identical extensive or semi-intensive management systems in the Assiut Governorate were enrolled, to eliminate environmental and management-related confounding effects.

### Clinical examination and laboratory measurement techniques

Data for each variable of interest were gathered through standardized clinical examinations and laboratory evaluations completed uniformly across all animal groups to ensure absolute comparability. Clinical Variables (Fever and Tick Infestation): Body temperature was taken rectally with a calibrated digital thermometer, and pyrexia was defined as a temperature over 39 °C. Tick infestation was determined through the physical examination of predilection locations (e.g., perineum, udder, ears) and quantified systematically. Parasitological Variables; Blood samples were taken from each animal’s jugular vein under aseptic circumstances. Two standardized procedures were used to detect *Babesia* spp.; the microscopic examination of Giemsa-stained thin blood smears under oil immersion lens to assess parasite morphology, and molecular detection using polymerase chain reaction (PCR) assays targeting particular parasite DNA.

### Blood sampling

Approximately 5 milliliters of blood were drawn from the jugular vein of each animal and placed in tubes containing ethylene-diamine tetraacetic acid (EDTA) [19]. The samples were dispatched in an icebox to the Animal Health Institute’s Assiut Regional Laboratory for molecular processing and parasitological analysis within a few hours of collection.

### Sample size

The minimum required sample size was determined using the Free Raosoft Sample Size Calculator (Raosoft Inc., Seattle, WA, USA; http://www.raosoft.com/samplesize.html), which is based on the epidemiological formula for cross-sectional research. Based on previous regional data [20], the predicted prevalence of *Babesia* spp. was set at 6.2%, with a 95% confidence interval (Z = 1.96) and a 5% absolute precision (d = 0.05). The software calculated that a minimum sample size of 89 animals was statistically necessary for this study. However, in order to increase the accuracy and dependability of the predicted prevalence, 200 animals in total were included in this study.

### Microscopic examination

Each sample was processed into thin blood smears and preserved in 100% methanol for two minutes after being allowed to air dry. The smears were stained with a 10% Giemsa solution and then cleaned with buffered water. The investigation was conducted using an Olympus microscope (XSZ-107BN series of Bio-Microscope, China) that was immersed in oil and magnified 100 times [21]. The criteria established by [22] were used to identify the erythrocytic forms of *Babesia* species.

### Molecular inquiry

#### Extraction of DNA

The QIAamp DNA Mini Kit (QIAGEN, Hilden, Germany, Cat. No. 51304) was used in accordance with the manufacturer’s instructions to extract genomic DNA from blood samples (*n* = 8) that demonstrated heavily infected microscopically.

#### PCR amplification

The 18 S rRNA gene was used to amplify *Babesia* spp. in compliance with established methods from previous studies [23, 24]. The PCR reaction mixture was 25 µL in volume and consisted of 12.5 µL of 2X PCR master mix, 5 µL of DNA template, 1 µL of each primer, and 5.5 µL of nuclease-free water. After a 5-minute initial denaturation at 94 °C, the PCR amplification was carried out in 35 cycles of secondary denaturation at 94 °C for 30 s, annealing at 55 °C for 40 s, and primary extension at 72 °C for 40 s. The last extension stage was performed at 72 °C for ten minutes. The PCR temperature, duration, and primer set are shown in Tables 1 and 2. Using Red SafeTM nucleic acid stain, the amplified products were separated on a 1.5% agarose gel. The DNA bands were observed under UV light using a gel documentation system, and the images were captured for examination.

**Table 1:** Target genes, nucleotide sequences, and amplicon sizes of the nominated primers

Target gene	Primers sequences	Amplified segment (bp)	Reference
*Babesia 18 S rRNA*	GTCTTGTAATTGGAATGATGGTGAC	340	[25]
	ATGCCCCCAACCGTTCCTATTA		

**Table 2:** Cycling conditions of the primers during cPCR

Gene	Primary denaturation	Sec. denaturation	Annealing	Extension	Final extension
		35 cycles			
*Babesia* *18 S rRNA*	94˚C 5 min.	94˚C 30 s.	55˚C 40 s.	72˚C 40 s.	72˚C 10 min.

### Sequencing and phylogenetic analyses

The PCR products from the samples that produced the clearest and most distinguishable bands (*n* = 5) were purified using the QIAquick PCR Product Extraction Kit (Qiagen, Valencia, CA, USA). The sequencing was done at the Molecular Biology Research Laboratory, Animal Health Research Institute, Cairo Branch, Egypt, using the BigDye Terminator v3.1 Cycle Sequencing Kit (PerkinElmer, Waltham, MA, USA). The reactions were cleaned using CentriSep spin columns, and DNA sequences were extracted using an Applied Biosystems 3130 genetic analyzer (ABI, 3130, USA). Genetic similarity was calculated by comparing the NCBI database (http://www.ncbi.nlm.nih.gov/) with the BLAST reference [26]. The sequences were compared using the CLUSTAL W multiple sequence alignment program, version 12.1 of the Meg Align module of Laser gene DNA Star Software Pairwise (Madison, Wisconsin, USA), which was developed by [27]. MEGA 6’s maximum likelihood, neighbor joining, and maximum parsimony were used for phylogenetic studies [28].

### Tick collection and identification

Curved forceps were used to physically remove engorged ticks from the cattle’s perennial area, ear, neck, thorax, abdomen, tail, udder, and forelegs. The removal was done carefully with a counterclockwise rotation to minimize damage to the specimens [29, 30]. The animals were separated into four groups based on their tick infestation and *Babesia* infection status: (1) infected and infested, (2) non-infected but infested, (3) infected but not infested, and (4) non-infected and non-infested. Ticks were stored in labeled Falcon tubes containing 70% ethanol and 30% glycerin for additional morphological identification. A stereomicroscope (SZ-ST Olympus, Japan) was used to inspect the ticks, and captured on integrated digital camera. The surveyed ticks were documented in accordance with [31].

#### Statistical analysis

The data were analyzed with SPSS software (version 9.5.1; IBM Corp., Armonk, NY, USA). Descriptive statistics, such as frequencies and percentages, were employed to express the prevalence of *Babesia* species. Initially, a univariate analysis using the Chi-square test (χ²) test was performed to analyze the statistical associations between individual risk factors (such as host age, gender, breed, and tick infestation status) and parasite positive. To compensate for potential confounding variables and find independent predictors of infection, all risk factors with a *P*-value < 0.20 in the univariate analysis were put into a multivariate logistic regression model. Adjusted odds ratios (aOR) and 95% confidence intervals (CI) were calculated. All statistical tests were considered statistically significant if the *P*-value was less than 0.05.

To assure the authenticity and reliability of the epidemiological data, numerous methods were implemented to reduce potential sources of bias: a random sample strategy was used to screen crossbred cattle across the study area, ensuring that animals of different ages (calves, young, and adults) had an equal chance of being examined, as opposed to convenience sampling. To reduce measurement and information bias, all clinical tests (such as rectal temperature and tick count) and laboratory analyses (microscopy and PCR) followed strictly established methods. Furthermore, to avoid observer bias, laboratory workers were blinded to the samples’ exact farm origin and clinical history during microscopic and molecular testing.

## Results

**Fig. 1:**
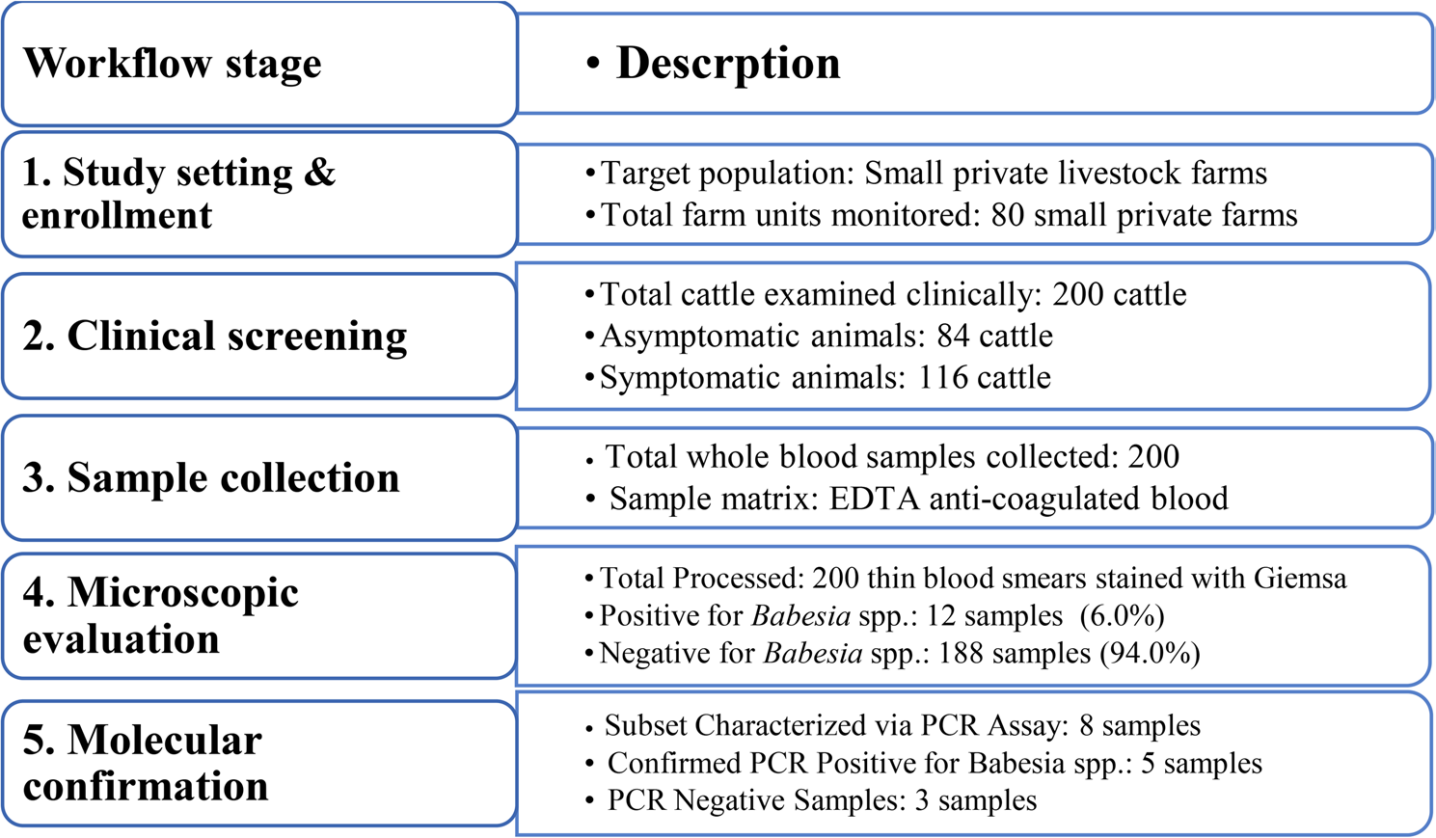
Flowchart detailing the sequential stages of herd recruitment, clinical evaluation, microscopic screening, and molecular PCR confirmation of *Babesia* spp. in cattle

The detailed sequential stages of herd recruitment, clinical screening, and laboratory molecular validation workflows are systematically illustrated in (Fig. 1).

### Prevalence of *Babesia* species in cattle under examination and related risk factors using a conventional microscope

According to the current inquiry, 6% (12/200) of the samples examined under a microscope for this study tested positive for *Babesia* spp. 8.6% (10/116) of the infected animals had symptoms of *Babesia* spp. infection, including fever, emaciation, congested mucous membrane, anemia, anorexia, and hemoglobinuria, while 2.4% (2/84) had no symptoms at all and tested positive for *Babesia* spp. using blood film analysis.

Regarding age as a potential risk factor, young animals under a year old exhibited a greater overall prevalence of *Babesia* spp. (8.75%) than adults over a year old, as Table 3 illustrates. According to sex, the total prevalence of *Babesia* spp. was higher in males (8.3%) than in females (4.3%). Regarding the seasonal variation, 4 (4.2%) of the 96 animals whose samples were collected in cold weather exhibited *Babesia* spp. infections, compared to 8 (7.7%) of the 104 animals whose samples were collected in hot weather. However, age, sex, or seasonal variation did not seem to be associated with the *Babesia* infection. Furthermore, *Babesia* spp. infection rates were significantly greater in cattle infested with hard ticks.

**Table 3:** shows the prevalence of *Babesia* infection in cattle by age, sex, season, tick infestation, and clinical signs

Varaible	No. exam.	No. of infected (%)	Ch^2^	*P*. value
Total	200	12 (6%)		
Clinical condition				
Asymptomatic	84	2 (2.4%)	2.35	0.125
symptomatic	116	10 (8.6%)		
Age				
< 1 year	80	7 (8.75%)	2.05	0.358
1–3 year	56	3 (5.4%)		
> 3 year	64	2 (3.12%)		
Gender				
Male	84	7 (8.33%)	0.78	0.378
Female	116	5 (4.3%)		
Season				
Hot	104	8 (7.7%)	0.56	0.453
Cold	96	4 (4.2%)		
Tick infestation				
Infected-infested	200	69 (34.5%)	52.02	0.0001^**^
Non-infected- infested	200	28 (14%)		
Infected-non infested	200	20 (10%)		
Non-infected-non infest.	200	83 (41.5%)		

**High Significant statistical difference

The risk factor analysis using multivariate logistic regression (Table 4) demonstrated that among all investigated variables, physical tick infestation was the primary, independent predictor of *Babesia* spp. infection in the examined population. In the univariate assessment, factors such as symptomatic clinical condition (Crude OR = 3.87; 95% CI: 0.83–18.06) and the hot season (Crude OR = 1.92; 95% CI: 0.55–6.65) displayed baseline descriptive variations. However, after incorporating all variables into the final multivariate logistic regression model to control for potential confounding effects, the statistical significance of these factors diminished (*P* > 0.05). Conversely, tick-infested animals maintained a remarkably powerful and statistically independent risk (Adjusted OR = 21.64; 95% CI: 2.35–199.12; *P* = 0.001).

**Table 4:** univariate and multivariate logistic regression analysis of potential risk factors associated with *Babesia* spp. infection in the examined herds (*N* = 200)

Risk factor	No. Exam.	No. Positive (%)	Crude OR (95% CI)	*P*. value	Adjusted OR (95% CI)	*P*. value
Clinical condition						
Asymptomatic	84	2 (2.4)	1.00 (Ref)		1.00 (Ref)	
Symptomatic	116	10 (8.6)	3.87 (0.83–18.06)	0.125	1.94 (0.32–11.75)	0.468
Age						
< 1 year	80	7 (8.75)	2.97 (0.59–14.82)	0.358	2.14 (0.41–11.18)	
1–3 year	56	3 (5.4)	1.78 (0.29–10.99)	0.536	1.32 (0.20–8.71)	
> 3 year	64	2 (3.12)	1.00 (Ref)		1.00 (Ref)	
Gender						
Male	84	7 (8.33)	2.02 (0.61–6.67)	0.378	1.68 (0.48–5.88)	0.418
Female	116	5 (4.3)	1.00 (Ref)		1.00 (Ref)	
Season						
Hot	104	8 (7.7)	1.92 (0.55–6.65)	0.453	1.45 (0.38–5.54)	0.584
Cold	96	4 (4.2)	1.00 (Ref)		1.00 (Ref)	
Tick infestation						
No	103	0	1.00 (Ref)	0.0001**	1.00 (Ref)	0.001**
Yes	97	12 (12.4)	29.85 (3.24–275.14)		21.64 (2.35–199.12)	

### Morphological examination of isolated *Babesia* species under a light microscope

**Fig. 2:**
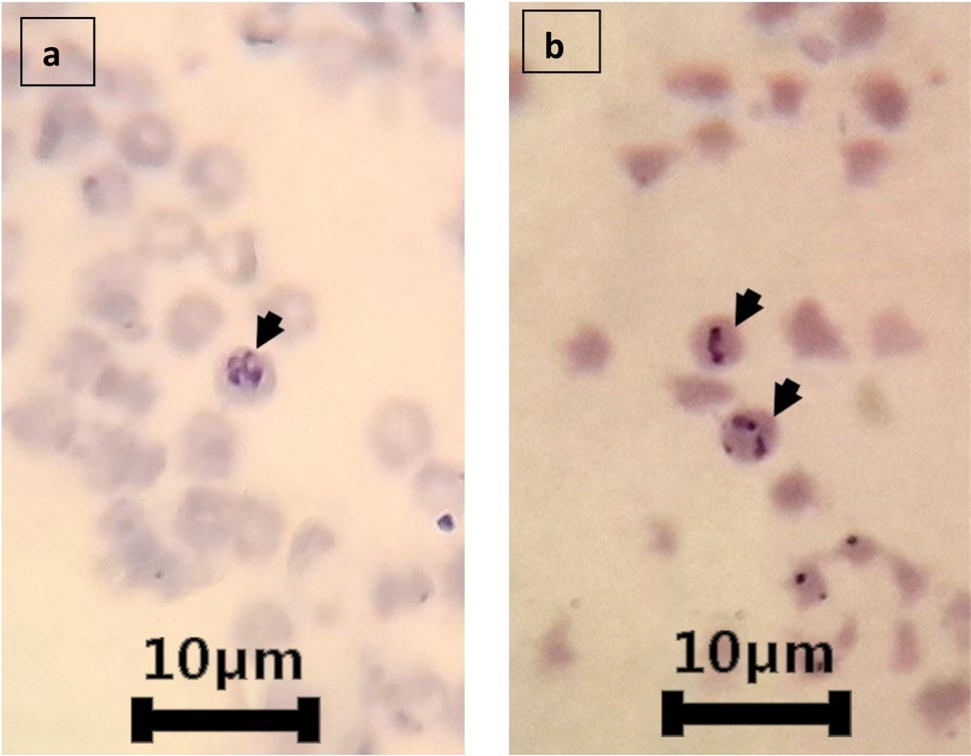
Blood smears stained with Geimsa from cattle with intraerythocytic piroplasm of (**a**) *B. bigemina* (black arrow head) and (**b**) *B. bovis* (black arrow head), scale bar 10 micron

Two species of intraerythrocytic piroplasm stages of *Babesia* spp. were revealed by microscopic examination of stained blood samples. The large, pyriform (pear-shaped) trophozoites of *B. bigemina* (Fig. 2, a) measure 4–5 mic. x 2–3 mic. Usually found in pairs, they are linked at acute angle. The organism has a tiny, darkly stained purple mass nucleus at the pole, and its cytoplasm is pale blue. The second species, *B. bovis* (Fig. 2, b), is characterized by tiny, pear-shaped, obtuse-angled trophozoites that are located in the core region of infected erythrocytes. These trophozoites have a length of 1.0 to 2 μm and a width of about 1.5 μm. *B. bovis* has a dark red nucleus and pale blue cytoplasm, and their chromatin is often found at one end of the parasite.

The complete cross-tabulation and diagnostic alignment between the initial microscopic screening and the confirmatory PCR results are summarized in (Table 5).

**Table 5:** Cross-tabulation of microscopic examination and confirmatory PCR results for *Babesia* detection (*N* = 200)

Microscopic exam.	PCR confirmed positive	PCR confirmed negative	Total
Positive	5	3	8 (PCR tested)
Positive	Not tested	Not tested	4 (PCR not tested)
Negative	Not tested	Not tested	188 (PCR not tested)
Total	5	3	200

### Molecular analysis

**Fig. 3:**
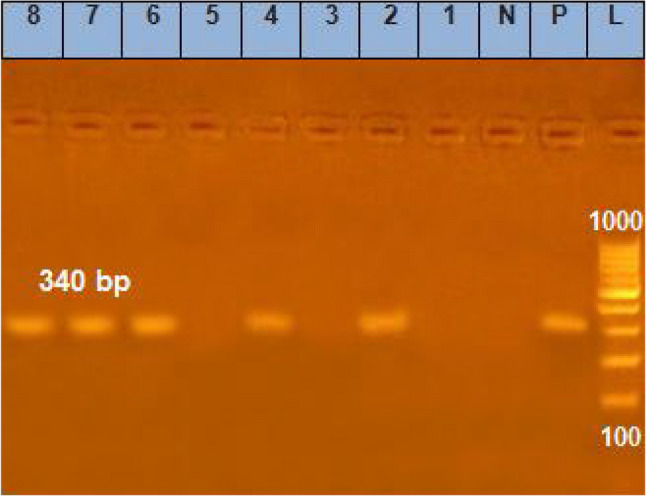
An agarose (1.5%) gel shows the 340 bp *Babesia* PCR products generated from positive samples. The positive sample is represented by lanes (2, 4, 6, 7, 8), the negative sample by lanes (1, 3, 5), the DNA size marker by lane (L), and the positive and negative controls for *Babesia* are represented by lanes (P) and (N), respectively

**Fig. 4:**
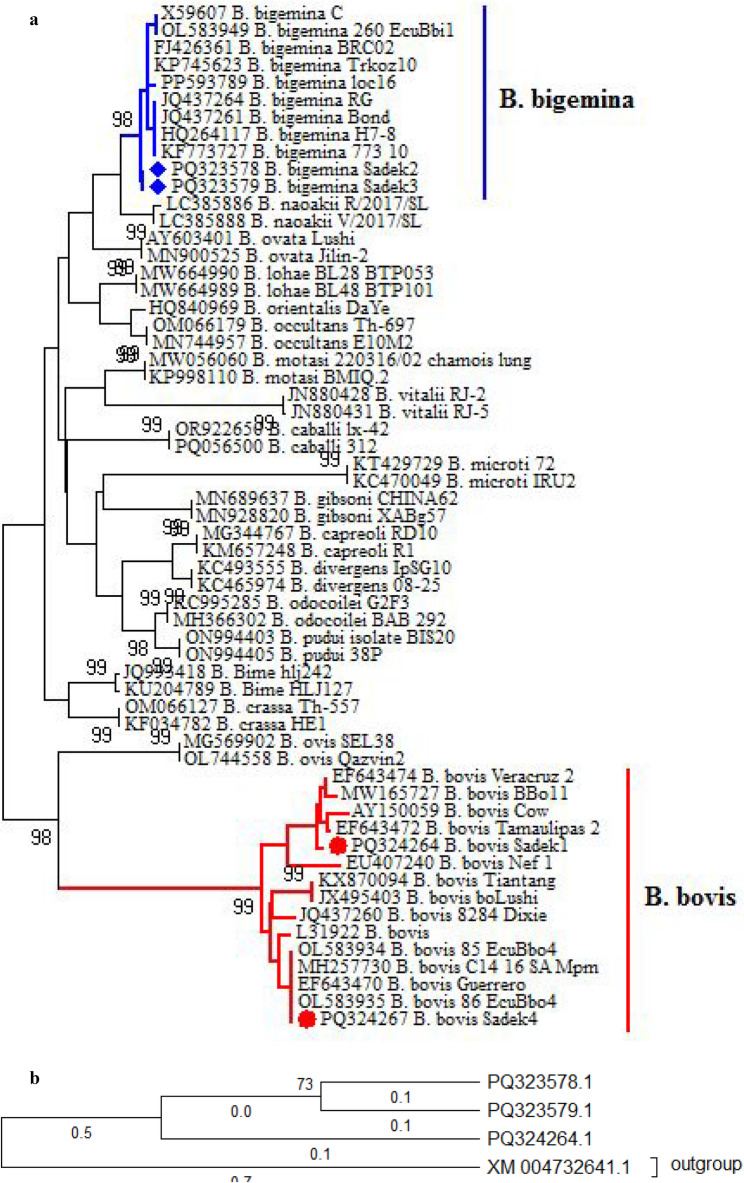
**a** The phylogenetic tree built using the 18 S rRNA gene sequences showed several clades that represent various genetic lineages of *Babesia bovis* and *Babesia bigemina*. The bar in the illustration represents the evolutionary distance divergence, which is 0.02 substitutions per site. **b**: Rooted Maximum Likelihood tree of the study isolates using *Theileria annulata* (XM_004732641.1) as an outgroup; PQ323578.1 and PQ323579.1 refer to our *Babesia bigemina* study isolates (Sadek2 and Sadek3, respectively), PQ324264.1 refers to our *Babesia bovis* study isolate (Sadek1). XM_004732641.1 represents the *Theileria annulata* sequence utilized as the outgroup to ensure proper phylogenetic routing. The numbers at the nodes represent the bootstrap confidence values (1,000 replicates), and the scale bar indicates the evolutionary distance

Molecular identification was used to confirm the results of microscopy analysis. Eight blood samples with a high parasite load under a microscope and a considerable level of parasitic infection were selected for PCR analysis. In these samples, *Babesia* spp. were found. Additionally, after *Babesia* 18 S rRNA was amplified using the PCR technique, 340 bp bands that were visible under UV light on a 1.5% agarose gel were found (Fig. 3). The amplified product’s ensuing sequencing confirmed our sample’s association with *B. bigemina* and *B. bovis*. The pertinent sequence can be found in GenBank under the accession codes PQ323578, PQ323579, and PQ324264, PQ324267 for *B. bigemina* and *B. bovis*, respectively (Fig. 4; a, b).

**Fig. 5:**
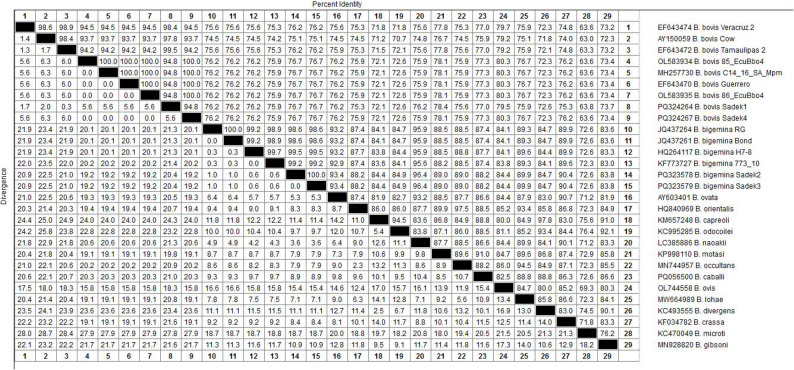
The 18 S rRNA gene determines the percentages of identity for the investigated isolates of *B. bigemina* and *B. ovis* in cattle compared to other isolates worldwide

Additionally, (Fig. 5) shows the percentages of identity between our isolate’s 18 S rRNA nucleotide sequences and those of 29 reference strains of *Babesia* species. The grouping nodes of the species under study are well supported by the high bootstrap values (100%) in the figure. The percentage support for the associations produced from the phylogenetic tree is reliably indicated by the bootstrap values.

### Morphological identification of isolated hard ticks

**Fig. 6:**
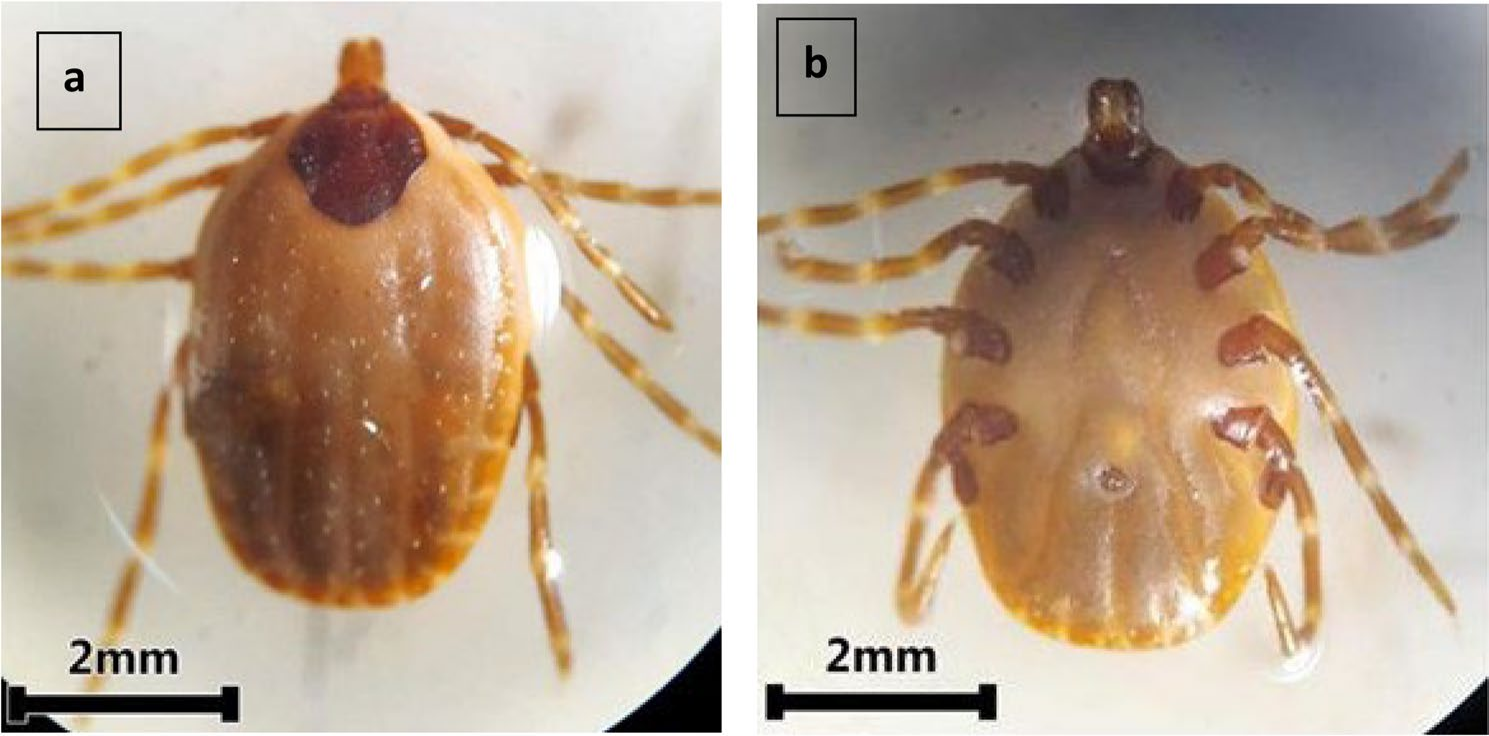
Adult female *Hyalomma* spp. hard ticks; **a** dorsal view, **b** ventral view; scale bar =2 mm

Tick infestations were found in 48.5% (97/200) of the cattle that were investigated. The adult tick has four pairs of legs with pale rings on both sexes, an anterior mouthpart, and flattened dorsoventrally. The unfed adult is 5–7 mm long, but the engorged female (Fig. 6; a- dorsal, b-ventral) is 15–18 mm. A small dark brown scutum only covers the front part of the female’s body on the dorsal side. The well-developed, comma-shaped spiracular plates are located between coxae II on the ventral side, where the vaginal opening is located.

**Fig. 7:**
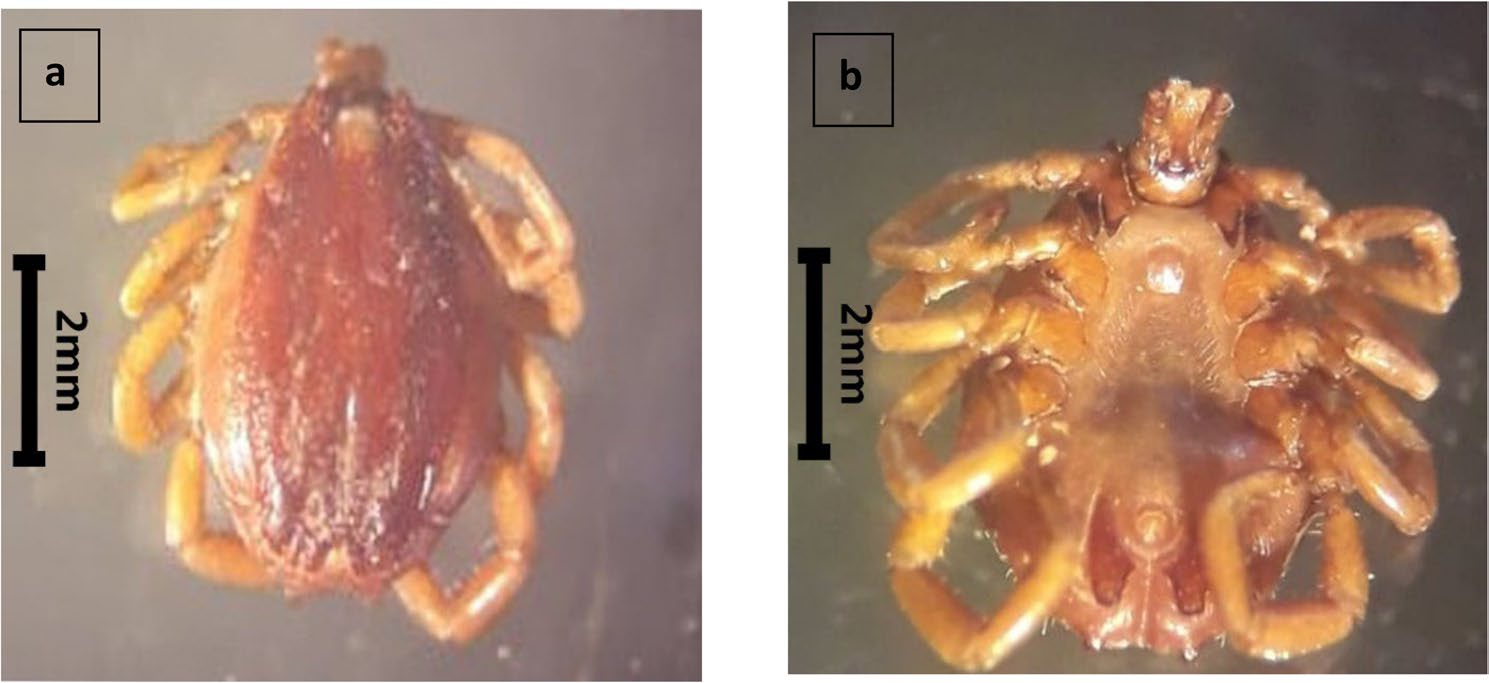
Adult male *Hyalomma* spp. hard ticks; **a** dorsal view, **b** ventral view; scale bar =2 mm

The male hard tick (Fig. 7; a-dorsal, b- ventral) has a glossy brown scutum that covers the entire dorsum and is often much smaller, measuring 4–6 mm. Male adanal plates are clearly visible and have square ventral ends. The basis capituli is rectangular dorsally, and both sexes have noticeable festoons along the posterior margin (the center festoon is pale in males). All of the earlier morphological characteristics were compatible with the *Hyalomma* species’ morphology.

## Discussion

Babesiosis is one of the illnesses that negatively impacts the health of our animals and the biggest barrier to successful production [32]. Globally, *B. bovis* and *B. bigemina* are particularly important economically [33]. Due to insufficient estimates of their occurrence and a lack of preventive actions, Egypt continues to overlook these ailments [34]. Thus, early and precise diagnosis of babesiosis is essential for managing and treating this severe disease.

In accordance with the study’s core aims, our investigation effectively produced a complete epidemiological and molecular mapping of *Babesia* illnesses in crossbred cattle in the Assiut Governorate.

First, in terms of the prevalence aim, our findings established a baseline infection rate, indicating a large health burden in the investigated group. Second, with regard to the molecular characterization objective, PCR and subsequent sequencing successfully identified the circulating species, demonstrating the active endemicity of *Babesia* spp. in Upper Egypt. Finally, in terms of risk factors, the study successfully correlated host-related variables. Collectively, these results directly fulfill the established study objectives, giving critical data for devising focused control strategies.

Microscopic analysis revealed that 6% of the crossbred cattle under study infected with *Babesia* spp. This finding was nearly identical to that of [35], who showed that 8.15% of Menofia had *Babesia* spp., and [36] in Bangladesh, who reported that 5.9% had *Babesia*. Even so, the findings were higher than those of [37] in India, who found 1.9% infected with *Babesia* spp., and [38] in Pakistan, who reported 0.22% and 0.44% infected with *B. bovis* and *B. bigemina* respectively. However, the current result was lower than that of [11] in the Qalyobia Governorate (22.47%) and [34] in the three major regions of Egypt (northern, central, and southern) (11.16%). This fluctuation in the prevalence of babesiosis in cattle may be caused by the location of tick vectors, breeds, weather, farm management, and sampling settings [39].

In terms of the clinical picture of animals infected with babesiosis, the current results revealed that 10 (8.62%) had symptoms specific to *Babesia* spp. infection, including fever, anorexia, congested mucous membrane, weakness, and hemoglobinuria, while 2.38% had no symptoms and tested positive for *Babesia* spp. using blood film analysis. Similar findings were reported by [40] and [10].

The current study found that the prevalence of *Babesia* infection was non-significantly greater in calves under one year (8.75%) than in older animals. Similar findings were obtained by [11, 41], and [42], who demonstrated that calves less than a year had a higher rate of *Babesia* spp. infection than adults. Our results, however, conflict with those of [34] and [42], who found that the prevalence was higher in older animals than in younger ones. Two possible causes for increased infectivity in young stock are tick mouth parts that can readily pierce the weaker skins of young animals and less or no exposure to the parasites, which weakens immunity [2].

Our study found that males had a non-significantly higher prevalence of *Babesia* (8.33%) than females (4.3%). The outcomes are in line with those of [43, 44], and [45]. Because they are housed inside, where a tick vector may infest them extensively, and they work in agriculture, which can lead to stress and weaken their immune systems, males may be more susceptible to contracting *Babesia* than females [44]. Breed, location, and management techniques are some of the variables that may affect these variations [46].

In regarding seasonal variations, the current study revealed that *Babesia* was more prevalent in the hot season (7.7%) than in the cold season (4.2%). The distribution by season varied very little. Many studies [47–49], and [50] came to similar conclusions, noting that babesiosis is more prevalent in hot months. This is due to the fact that high temperatures greatly rise the dispersal of hard ticks, the main vector of *Babesia* spp [51, 52].

Regarding the relationship between *Babesia* infection prevalence and tick infestation. *Babesia* infection rates in cattle infested with hard ticks were much higher than those of other groups, with a prevalence rate of 34.5%. The current research supports the findings of [48, 53], and [49], which validate the role of ticks as vectors in the transmission of these illnesses across animal hosts.

In order to differentiate between *B. bigemina* and *B. bovis*, our work amplified and sequenced the 18 S rRNA from a blood sample that tested positive for *Babesia*. The sequence was then added to GenBank. A significant bootstrap value, which highlights the potential of these gene sequences for accurately identifying the isolated *Babesia* from a positive blood sample, is supported by the study’s phylogenetic analysis. Our understanding of the geographical spread of *Babesia* spp. is expanded by this new finding. The 18 S rRNA was also utilized by [54, 55], and [42] to verify the presence of babesiosis in cattle. Similar findings have been reported by [33, 56], and [57] in various Egyptian governorates, indicating *B. bigemina* as the most prevalent strain.

Although this work presents epidemiological and molecular evidence, some limitations must be highlighted. Firstly; to address selection bias, the screening focused on crossbred cattle exhibiting clinical symptoms (pyrexia, hemoglobinuria, or tick infestation). This method may add a positive directional bias, perhaps overestimating the true population-level prevalence of *Babesia* spp. in the area. However, the severity of this bias was reduced by using a random selection procedure during the initial field screening. Secondly, in terms of diagnostic imprecision, using standard light microscopy for initial screening has a negative directional bias since it consistently misses subclinical infections or cases with low parasitemia (underestimation). Furthermore, due to financial and logistical limitations, molecular PCR validation was restricted to a selection of samples rather than the complete cohort. Additionally, the molecular screening of the collected vectors for *Babesia* spp. was not performed. Future large-scale longitudinal research are highly recommended. Such studies should include entirely asymptomatic herds, larger cohorts for simultaneous host-vector PCR screening, and extensive sequencing to provide a more definitive epidemiological mapping of the parasite’s transmission dynamics and genetic diversity in the region.

## Conclusion

Recent research indicates that *Babesia* is still a common and dangerous blood parasite that seriously impairs Egyptian cow health. Our investigation, which included PCR and microscopic examination to detect bovine babesiosis, discovered two species of *Babesia (B. bovis and B. bigemina)* in crossbred cattle in the Assiut Governorate of Upper Egypt. To halt the disease’s spread, effective control measures are desperately needed. These include treating affected animals in particular and implementing all-encompassing programs to eradicate ticks from cattle and their surroundings.

## Supplementary Information

Supplementary Material 1.

Supplementary Material 2.

Supplementary Material 3.

## Data Availability

The 18 S rRNA gene sequences generated and analyzed during the current study have been deposited in the NCBI Gen Bank database:- *Babesia bovis* 18 S rRNA sequences are available under accession numbers PQ324264, PQ324267 and can be accessed at: [https://submit.ncbi.nlm.nih.gov/subs/?search=SUB14726398](https:/submit.ncbi.nlm.nih.gov/subs/?search=SUB14726398) [https://submit.ncbi.nlm.nih.gov/subs/?search=SUB14726412](https:/submit.ncbi.nlm.nih.gov/subs/?search=SUB14726412)- *Babesia bigemina* 18 S rRNA sequences are available under accession numbers PQ323578, PQ323579 and can be accessed at: [https://submit.ncbi.nlm.nih.gov/subs/?search=SUB14726352](https:/submit.ncbi.nlm.nih.gov/subs/?search=SUB14726352).

## References

[1] Mahmmod YS. Molecular detection of natural Babesia bovis infection from clinically infected and apparently healthy water buffaloes (Bubalus bubalis) and crossbred cattle. J Buffalo Sci. 2012;1:55–60. 10.6000/jbs.v1i1.95.

[2] Siddique RM, Sajid MS, Iqbal Z, Saqib M. Association of different risk factors with the prevalence of babesiosis in cattle and buffalos. Pakistan J Agricultural Sci. 2020;57(2). 10.21162/PAKJAS/19.8626.

[3] Bock RJL, de Vos A, Jorgensen W. Babesiosis of cattle. Parasitology 2004, 129:Suppl:S247–269. 10.1017/s0031182004005190 10.1017/s0031182004005190 15938514

[4] Savić S, Vidić B, Grgić Z, Potkonjak A, Spasojevic L. Emerging vector-borne diseases–incidence through vectors. Front public health. 2014;2:267. 10.3389/fpubh.2014.00267. 25520951 10.3389/fpubh.2014.00267 PMC4251170

[5] Fu B-K, Tang T, Yue M, Chen J-J, Su H, Huang X-B, Yang Y-F, Hay SI, Fang L-Q, Liu W. Mapping the Global Distribution of Babesia Infections. Transbound Emerg Dis. 2025;2025(1):5889219. 10.1155/tbed/5889219. 41333613 10.1155/tbed/5889219 PMC12668841

[6] Sivakumar T, Igarashi I, Yokoyama N. Babesia ovata: Taxonomy, phylogeny and epidemiology. Vet Parasitol. 2016;229:99–106. 10.1016/j.vetpar.2016.10.006. 27809988 10.1016/j.vetpar.2016.10.006

[7] Ibrahim A, El Behairy A, Mahran K, Awad W. Clinical and laboratory diagnosis of piroplasmids in naturally infected cattle in Egypt. J Egypt vet med Assoc. 2009;69(2):197–209.

[8] Florin-Christensen M, Suarez CE, Rodriguez AE, Flores DA, Schnittger L. Vaccines against bovine babesiosis: where we are now and possible roads ahead. Parasitology. 2014;141(12):1563–92. 10.1017/S0031182014000961. 25068315 10.1017/S0031182014000961

[9] Azevedo BT, de Oliveira HN, Katiki LM, Vercesi Filho AE, Domingos AG, Antunes S, Okino CH, de Sena Oliveira MC, Ibelli AMG, Giglioti R. A small proportion of Zebu genetic background in crossbred calves may not be enough to improve resistance against natural bovine Babesia spp. infections. Vet Parasitol. 2024;328:110165. 10.1016/j.vetpar.2024.110165. 38490159 10.1016/j.vetpar.2024.110165

[10] Abdel-Hamied E, Arafa W, Mahmoud MM. Oxidative stress, hemogram, hepatorenal function evaluation and molecular diagnosis of babesiosis in crossbred cows naturally infected with B. bigemina. Adv Anim Vet Sci. 2020;8(12):1402–9. 10.17582/journal.aavs/2020/8.12.1402.1409.

[11] Elmoghazy HMEM, Abdelwahab MG, El-Sayed AA. Epidemiological studies on bovine babesiosis and theileriosis in Qalubia governorate. BVMJ. 2014;27:36–48.

[12] El-Dakhly KM, Arafa WM, Soliman S, Abdel-Fatah OR, Wahba AA, Esteve-Gasent MD, Holman PJ. Molecular detection, phylogenetic analysis, and genetic diversity of Theileria annulata, Babesia bigemina, and Anaplasma marginale in cattle in three districts of Egypt. Acta Parasitol. 2020;65(3):620–7. 10.2478/s11686-020-00189-z. 32207056 10.2478/s11686-020-00189-z

[13] Buling A, Criado-Fornelio A, Asenzo G, Benitez D, Barba-Carretero J, Florin-Christensen M. A quantitative PCR assay for the detection and quantification of Babesia bovis and B. bigemina. Veterinary parasitology 2007, 147(1–2):16–25. 10.1016/j.vetpar.2007.03.031 10.1016/j.vetpar.2007.03.031 17466458

[14] Ibrahim HM, Galon EMS, Tumwebaze MA, Byamukama B, Liu M, Mohammed-Geba K, Sheir SK, Galal-Khallaf A, Latif HMAE, Morsi DS. Serological Survey of Babesia bigemina and Babesia bovis in cattle and water buffaloes from Menoufia Province. Egypt Acta Parasitol. 2021;66(4):1458–65. 10.1007/s11686-021-00338-y. 34043120 10.1007/s11686-021-00338-y

[15] Mahmoud MS, Kandil OM, Nasr SM, Hendawy SH, Habeeb SM, Mabrouk DM, Silva MG, Suarez CE. Serological and molecular diagnostic surveys combined with examining hematological profiles suggests increased levels of infection and hematological response of cattle to babesiosis infections compared to native buffaloes in Egypt. Parasites vectors. 2015;8(1):319. 10.1186/s13071-015-0928-9. 26062684 10.1186/s13071-015-0928-9 PMC4467044

[16] Abdel-Shafy S, Abdullah HH, Elbayoumy MK, Elsawy BS, Hassan MR, Mahmoud MS, Hegazi AG, Abdel-Rahman EH. Molecular epidemiological investigation of piroplasms and anaplasmataceae bacteria in Egyptian domestic animals and associated ticks. Pathogens. 2022;11(10):1194. 10.3390/pathogens11101194. 36297251 10.3390/pathogens11101194 PMC9609901

[17] AHMED AA, SAYED FG, GALAL LA, ISMAIL T, GABER MM. Detection of parasites contaminating raw consumable vegetables in Assiut city, Assiut governorate, Egypt. J Egypt Soc Parasitol. 2020;50(3):557–64. 10.21608/jesp.2020.131086.

[18] Abdel-Rady A, Karmi M, Youssef M, Abdel-Raouf A, Madkour B. Some epidemiological studies on Theileria annulata infection in egypt. Res J Vet Pract. 2023;11(1):7–12. 10.17582/journal.rjvp/2023/11.1.7.12.

[19] Gupta SK, Singla LD. Diagnostic trends in parasitic diseases of animals, editors. Veterinary Diagnostics: Current Trends. Delhi.: Satish Serial Publishing House; 2012.

[20] KAMEL FA, DYAB AK, Abd-Elrahman KHEDRAA. Epidemiological and clinical manifestations of blood parasitic infections in cattle in Assiut Governorate Egypt. Assiut Veterinary Med J. 2024;70(181):8–16. 10.21608/avmj.2024.246898.1203.

[21] Saleh EM, El-Ella A, Ghada A, Al-Hosary AA. Molecular Epidemiological Studies on Tick-borne Pathogens in Cattle and Buffaloes in Assiut Governorates. J Appl Mol Biology. 2026;4(1):1–17. 10.21608/jamb.2025.380493.1047.

[22] Levine N. Veterinary Protozoology. Ames: In.: Iowa State Univ; 1985. pp. 130–232.

[23] Barghash SM. Molecular prevalence and phylogeny of some tick-borne parasites in ruminants in Sinai Peninsula, Egypt. Eur J Biomed Pharm Sci. 2022;9(3):15–25.

[24] Kumar B, Maharana BR, Thakre B, Brahmbhatt NN, Joseph JP. 18S rRNA gene-based piroplasmid PCR: an assay for rapid and precise molecular screening of Theileria and Babesia species in animals. Acta Parasitol. 2022;67(4):1697–707. 10.1007/s11686-022-00625-2. 36178614 10.1007/s11686-022-00625-2 PMC9523193

[25] Salem N, Farag H. Clinical, hematologic, and molecular findings in naturally occurring Babesia canis vogeli in Egyptian dogs. Veterinary Med Int. 2014;2014(1):270345. 10.1155/2014/270345. 10.1155/2014/270345 PMC3944204 24693460

[26] Altschul SF, Gish W, Miller W, Myers EW, Lipman DJ. Basic local alignment search tool. J Mol Biol. 1990;215(3):403–10. 10.1016/S0022-2836(05)80360-2. 2231712 10.1016/S0022-2836(05)80360-2

[27] Thompson JD, Higgins DG, Gibson TJ. CLUSTAL W: improving the sensitivity of progressive multiple sequence alignment through sequence weighting, position-specific gap penalties and weight matrix choice. Nucleic Acids Res. 1994;22(22):4673–80. 10.1093/nar/22.22.4673. 7984417 10.1093/nar/22.22.4673 PMC308517

[28] Tamura K, Stecher G, Peterson D, Filipski A, Kumar S. MEGA6: molecular evolutionary genetics analysis version 6.0. Mol Biol Evol. 2013;30(12):2725–9. 10.1093/molbev/mst197. 24132122 10.1093/molbev/mst197 PMC3840312

[29] Youssef SY, Yasien S, Mousa WMA, Nasr SM, El-Kelesh EAM, Mahran KM, Abd-El-Rahman AH. Vector identification and clinical, hematological, biochemical, and parasitological characteristics of camel (Camelus dromedarius) theileriosis in Egypt. Trop Anim Health Prod 2015, 47(4):649–56. 10.1007/s11250-015-0771-1 10.1007/s11250-015-0771-1 25677167

[30] ESSA AM, KOTB SA, HUSSEIN MK, DYAB AK. Epidemiological and morphological studies on Hyalomma Species infesting dromedary camels In Aswan governorate, Egypt. J Egypt Soc Parasitol. 2022;52(1):123–32.

[31] Walker AR. Ticks of domestic animals in Africa: a guide to identification of species. Volume 74. Bioscience Reports Edinburgh; 2003.

[32] Mohammed E, Elshahawy I. The current prevalence of bovine babesiosis and theileriosis infection in Egypt. Clin Med Images Int J. 2017;1(1):306–11. 10.15406/jbmoa.2018.06.00224.

[33] Ibrahim HM, Moumouni PFA, Mohammed-Geba K, Sheir SK, Hashem IS, Cao S, Terkawi MA, Kamyingkird K, Nishikawa Y, Suzuki H. Molecular and serological prevalence of Babesia bigemina and Babesia bovis in cattle and water buffalos under small-scale dairy farming in Beheira and Faiyum Provinces. Egypt Veterinary Parasitol. 2013;198(1–2):187–92. 10.1016/j.vetpar.2013.08.028. 10.1016/j.vetpar.2013.08.028 24075417

[34] Rizk MA, Salama A, El-Sayed SA-E-S, Elsify A, El-Ashkar M, Ibrahim H, Youssef M, El-Khodery S. Animal level risk factors associated with Babesia and Theileria infections in cattle in Egypt. Acta Parasitol. 2017;62(4):796–804. 10.1515/ap-2017-0096. 29035848 10.1515/ap-2017-0096

[35] Nayel M, El-Dakhly KM, Aboulaila M, Elsify A, Hassan H, Ibrahim E, Salama A, Yanai T. The use of different diagnostic tools for Babesia and Theileria parasites in cattle in Menofia. Egypt Parasitol Res. 2012;111(3):1019–24. 10.1007/s00436-012-2926-6. 22543747 10.1007/s00436-012-2926-6

[36] Hossain MJ, Raut S, Singh RP, Mishra P, Hossain MS, Dey AR, Kabir A, Anisuzzaman, Talukder MH, Shahiduzzaman M. Molecular detection of Babesia and Theileria from crossbred cattle in Sirajganj and Rangpur districts of Bangladesh. Veterinary Med Sci. 2023;9(2):899–906. 10.1002/vms3.989. 10.1002/vms3.989 PMC10029904 36331989

[37] Fatima SA, Gonuguntla HN, Muthappa PN, Sarangi LN. Molecular detection of Anaplasma, Babesia, Theileria, and Trypanosoma infection in cattle and buffaloes in India. J Parasitic Dis. 2024;48(3):450–9. 10.1007/s12639-024-01673-3. 10.1007/s12639-024-01673-3 PMC11319688 39145369

[38] Ahmed Z, Ali A, Waqas M, Ahmed I, Anwar N, Malik MI, Jabbar A, Hussain A, Nawaz M. Molecular identification and characterization of piroplasms infecting cattle in Azad Kashmir. Pak Vet J 2024, 44(3):611–8. 10.29261/pakvetj/2024.112

[39] Fereig RM, Mohamed SG, Mahmoud HY, AbouLaila MR, Guswanto A, Nguyen T-T, Mohamed AEA, Inoue N, Igarashi I, Nishikawa Y. Seroprevalence of Babesia bovis, B. bigemina, Trypanosoma evansi, and Anaplasma marginale antibodies in cattle in southern Egypt. Ticks and tick-borne diseases 2017, 8(1):125–31. 10.1016/j.ttbdis.2016.10.008 10.1016/j.ttbdis.2016.10.008 27789159

[40] EL-DIASTY MM, RASHEED NM, KHEDER ZA, ABD EL-MAGED, RR. Biochemical, pathological and molecular studies on babesiosis in calves. Egypt J Agricultural Res. 2017;95(3):1269–83. 10.21608/ejar.2017.150272.

[41] Zulfiqar S, Shahnawaz S, Ali M, Bhutta AM, Iqbal S, Hayat S, Qadir S, Latif M, Kiran N, Saeed A. Detection of Babesia bovis in blood samples and its effect on the hematological and serum biochemical profile in large ruminants from Southern Punjab. Asian Pac J Trop Biomed. 2012;2(2):104–8. 10.1016/S2221-1691(11)60202-5. 23569878 10.1016/S2221-1691(11)60202-5 PMC3609249

[42] El-Bahy NM, Menshawy SM, Goda WM, Nasr SM, AbouLaila MR, Bazh EK, Abou-Rwash AA. Molecular detection of Babesia bigemina and Babesia bovis in cattle in Behaira Governorate. Ejpmr. 2018;5(12):441–6.

[43] Idris M, Mohammed S, Bashar A, Ibrahim M. Cross-sectional study of cattle Babesiosis and associated risk factors in Nyala, South Darfur, Sudan. Tanzan Veterinary J. 2018;33(2):1–10. 10.4314/TVJ.V33I2.

[44] Fesseha H, Mathewos M, Eshetu E, Tefera B. Babesiosis in cattle and ixodid tick distribution in Dasenech and Salamago Districts, southern Ethiopia. Sci Rep. 2022;12(1):6385. 10.1038/s41598-022-10416-4. 35430623 10.1038/s41598-022-10416-4 PMC9013365

[45] Mahmood SL, Ahmed RB, Kakarash NA, Niranji SS, Ismael DO, Sheikh MOB. Microscopic and molecular studies of bovine Babesiosis in Sulaymaniyah, Iraq. Veterinary Parasitology: Reg Stud Rep. 2025;57(101192). 10.1016/j.vprsr.2024.101192. 10.1016/j.vprsr.2024.101192 39855877

[46] Nyabongo L, Kanduma EG, Bishop RP, Machuka E, Njeri A, Bimenyimana AV, Nkundwanayo C, Odongo DO, Pelle R. Prevalence of tick-transmitted pathogens in cattle reveals that Theileria parva, Babesia bigemina and Anaplasma marginale are endemic in Burundi. Parasites vectors. 2021;14(1):6. 10.1186/s13071-020-04531-2. 33402225 10.1186/s13071-020-04531-2 PMC7786990

[47] Patel M, Kumar N, Rathod P, Tyagi K, Sorathiya L. Incidence and Haematological changes in haemoprotozoan infections in bovines of South Gujarat. Ind J Vet Sci Biotechnol. 2017;13(2). 10.21887/ijvsbt.v13i2.10054.

[48] Masih A, Rafique A, Jabeen F, Naz S. Molecular epidemiology of bovine babesiosis in Punjab, Pakistan. Acta Sci Veterinariae. 2021;49. 10.22456/1679-9216.111565.

[49] Yousef SG, Sobhy NM, Gouda H, Emam MH. Sero epidemiological study on bovine babesiosis in cattle and buffaloes in Sharkia Governorate, Egypt. Open Veterinary J. 2024;14(7):1577. 10.5455/OVJ.2024.v14.i7.7. 10.5455/OVJ.2024.v14.i7.7 PMC11338614 39175968

[50] Abdelwahab KH, Tolba ME, Senosy W, Ahmed AM, Khedr AA, Abdelaziz K, Aboelhadid SM, Mahmoud WG. Comparative Diagnostic Efficacy of Microscopy, LAMP and PCR for Detection of Bovine Babesiosis in New Valley Governorate, Egypt. Acta Parasitol. 2026;71(1):19. 10.1007/s11686-025-01194-w. 41528597 10.1007/s11686-025-01194-w PMC12799746

[51] Rony S, Mondal M, Begum N, Islam M, Affroze S. Epidemiology of ectoparasitic infestations in cattle at Bhawal forest area, Gazipur. Bangladesh J veterinary Med. 2010;8(1):27–33. 10.3329/bjvm.v8i1.7399.

[52] Roy A, Rahman M, Majumder S, Sarker A. Ecology of ticks and tick-borne blood protozoa in Modhupur forest area, Tangail. 2000.

[53] Farooq R, Hafeez MA, Oneeb M, Rafique A, Ashraf K, Aslam F, Rauf N, Khalid K, Bilal F, Mahmood S. Molecular characterization and phylogenetic analysis of Babesia species isolated from domestic cattle. 2020. 10.29261/pakvetj/2019.003

[54] Chaudhry ZI, Suleman M, Younus M, Aslim A. Molecular detection of Babesia bigemina and Babesia bovis in crossbred carrier cattle through PCR. Pakistan J Zool 2010, 42(2). https://www.zsp.com.pk/pdf/201-204%20%2813%29.pdf

[55] El-Fayomy AO, Ghoneim AM, Abu-Samak OA, Khidr AA. Contribution of Babesia to the illness of cows in Port Said Governorate, Egypt. Global Vet. 2013;11(1):118–22. 10.5829/idosi.gv.2013.11.1.7453.

[56] Adham F, Abd-El-Samie E, Gabre R, Hussein HE. Detection of tick blood parasites in Egypt using PCR. 2009. 10.1007/s00436-009-1443-8 10.1007/s00436-009-1443-8 19415329

[57] Al-Hosary A, Răileanu C, Tauchmann O, Fischer S, Nijhof AM, Silaghi C. Epidemiology and genotyping of Anaplasma marginale and co-infection with piroplasms and other Anaplasmataceae in cattle and buffaloes from Egypt. Parasites vectors. 2020;13(1):495. 10.1186/s13071-020-04372-z. 32993778 10.1186/s13071-020-04372-z PMC7526245

