# Supplementary figures and images for: An updated assessment of the molecular prevalence and risk factors of Babesia infection among crossbred cattle: a diagnostic cross-sectional study
Source: BMC Vet Res. 2026 Jun 26;22:367. doi: 10.1186/s12917-026-05639-w (PMC13309957; doi:10.1186/s12917-026-05639-w)

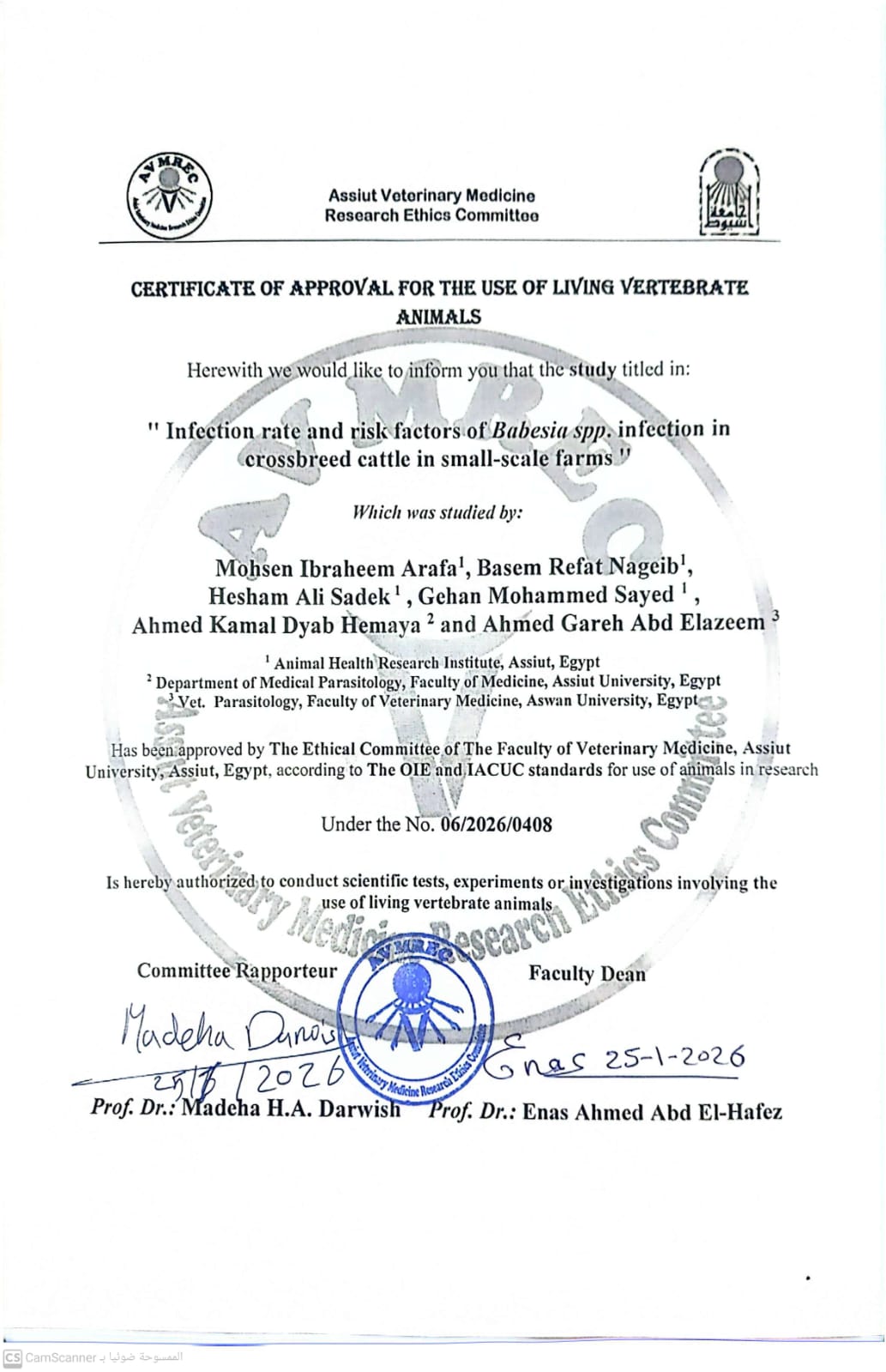

Supplement: Supplementary file 1 — Supplementary Material 1. [file 12917_2026_5639_MOESM1_ESM.jpeg]

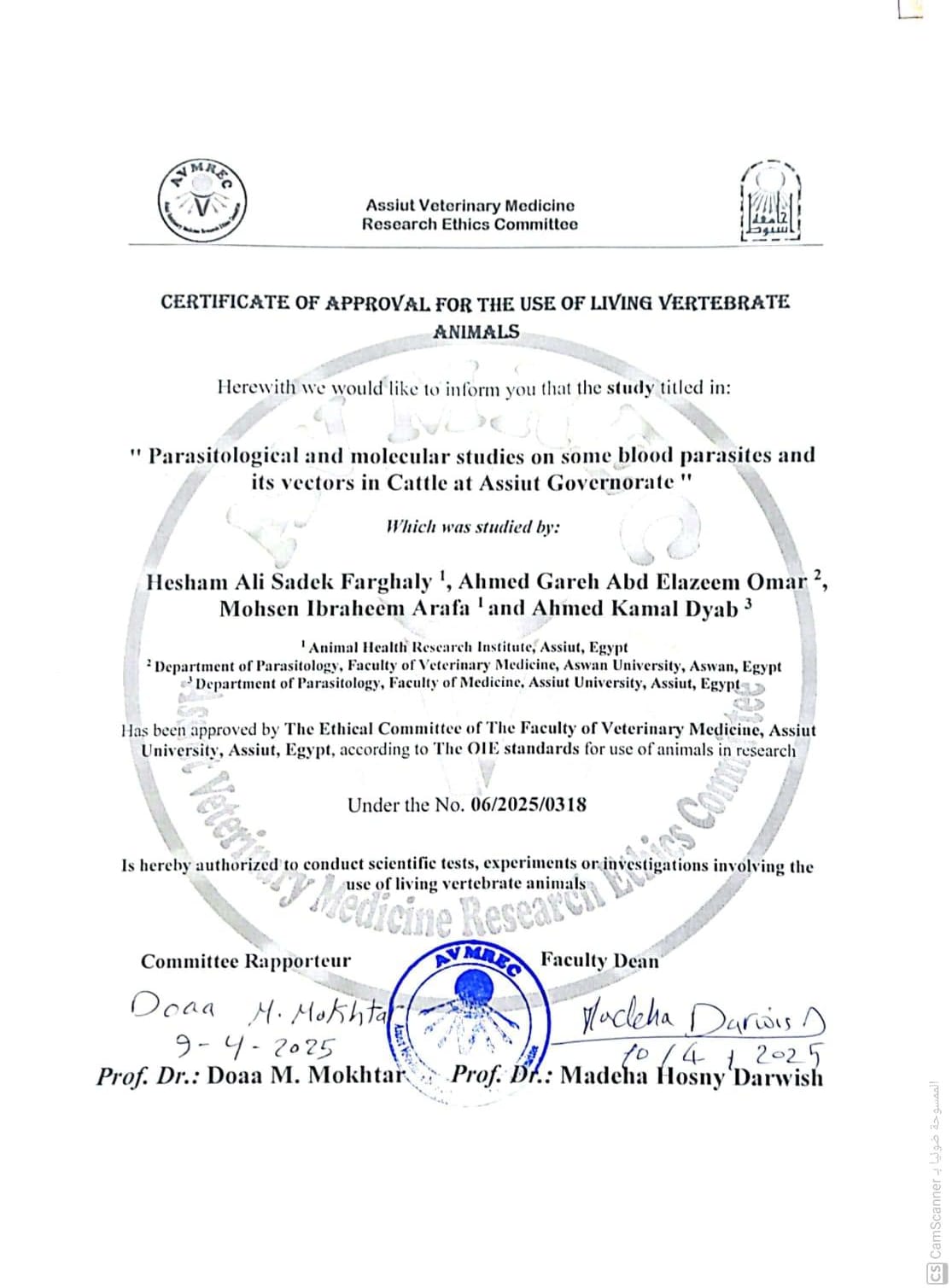

Supplement: Supplementary file 2 — Supplementary Material 2. [file 12917_2026_5639_MOESM2_ESM.jpg]
