# Supplementary material for: An updated assessment of the molecular prevalence and risk factors of Babesia infection among crossbred cattle: a diagnostic cross-sectional study
Source: BMC Vet Res. 2026 Jun 26;22:367. doi: 10.1186/s12917-026-05639-w (PMC13309957; doi:10.1186/s12917-026-05639-w)
Supplement: Supplementary file 3 — Supplementary Material 3. [file 12917_2026_5639_MOESM3_ESM.docx]

**
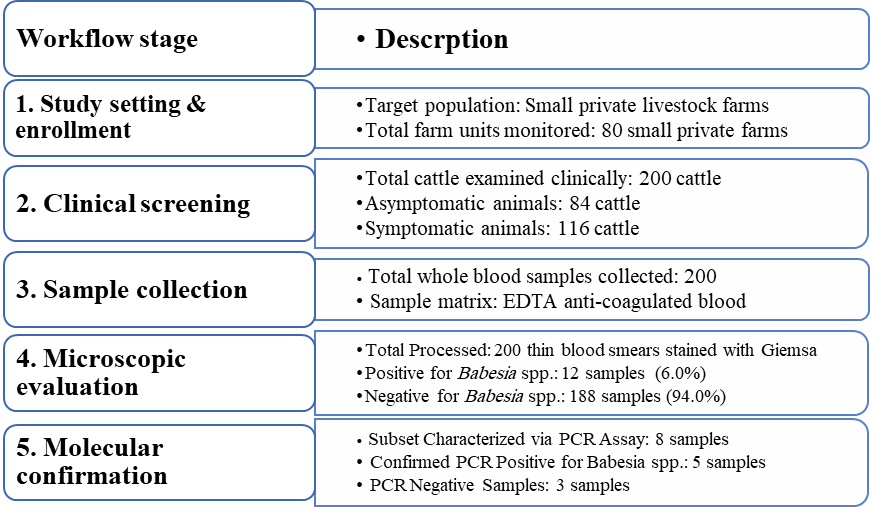
**

Figure 1: Flowchart detailing the sequential stages of herd recruitment,

clinical evaluation, microscopic screening, and molecular PCR confirmation of *Babesia* spp. in cattle.

**
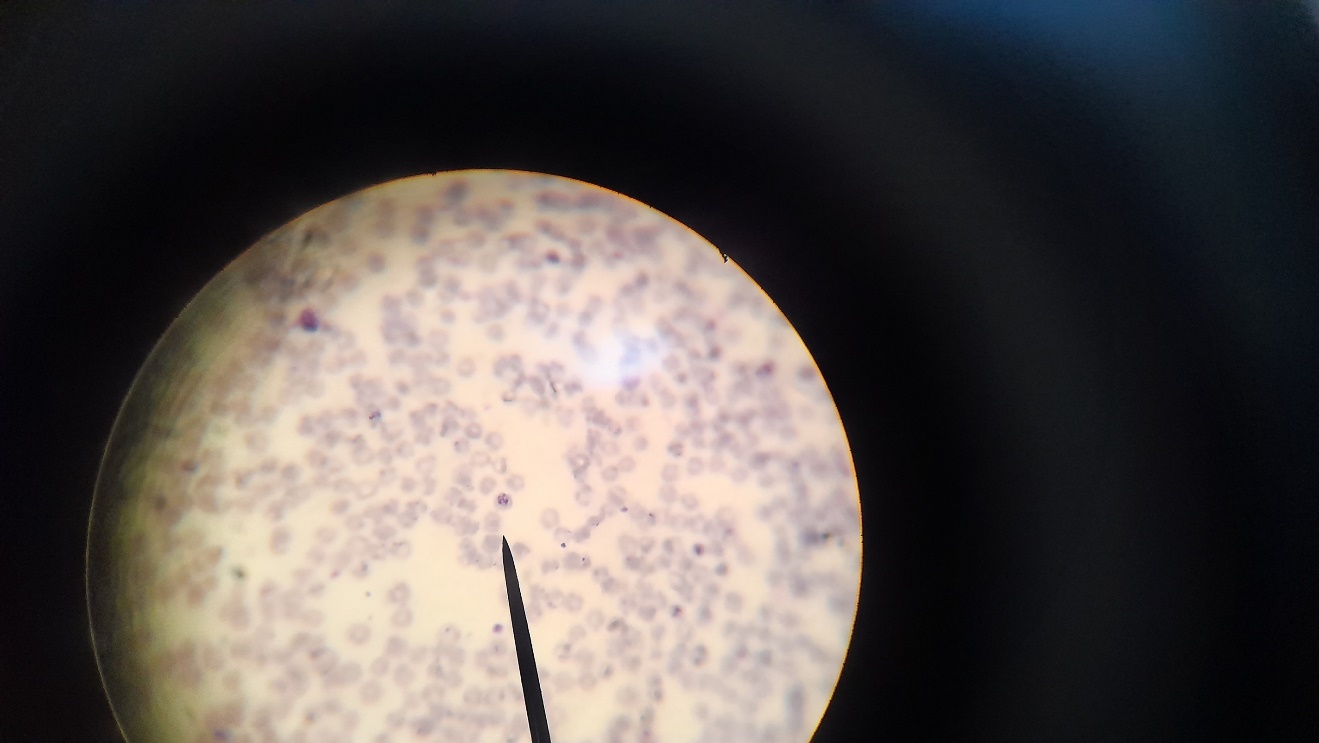
**

**Fig.2, a;** Blood smears stained with Geimsa from cattle with intraerythocytic piroplasm of *B. bigemina* (black arrow head).


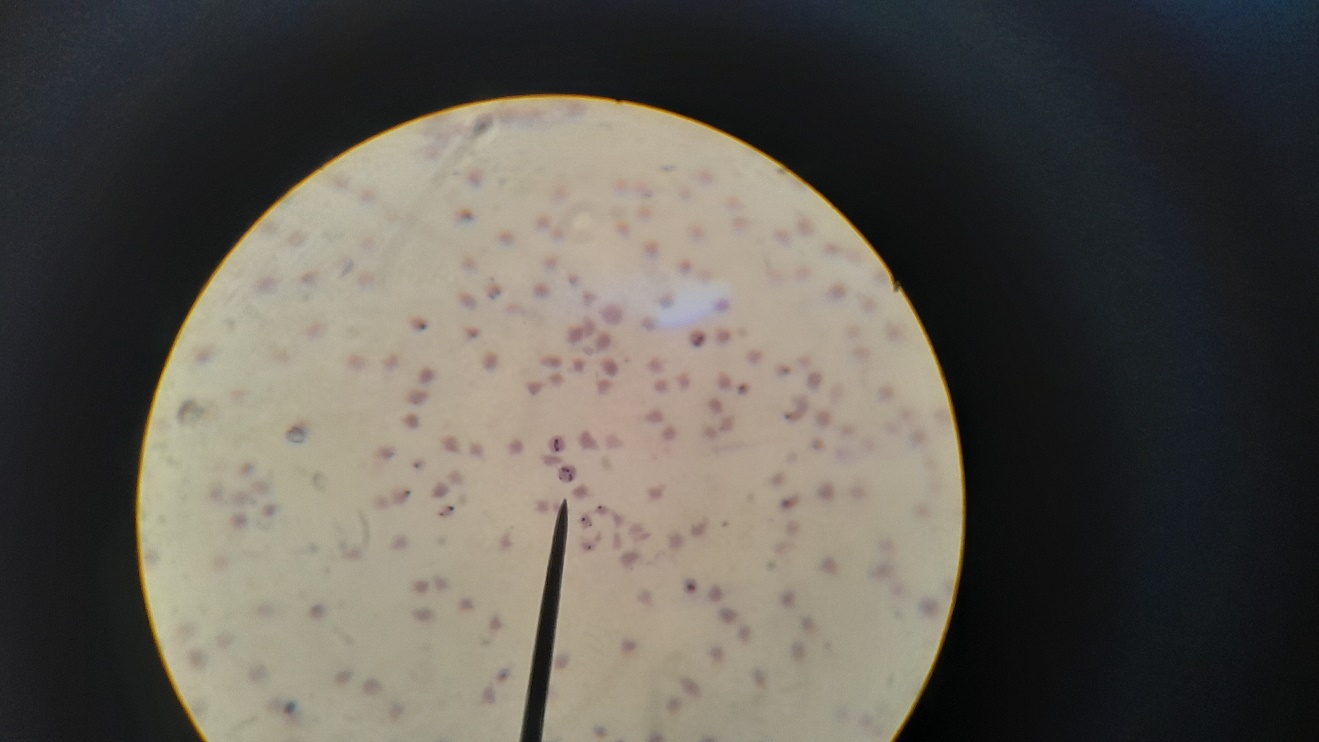


**Fig.2, b;** Blood smears stained with Geimsa from cattle with intraerythocytic piroplasm of *B. bovis* (black arrow head)


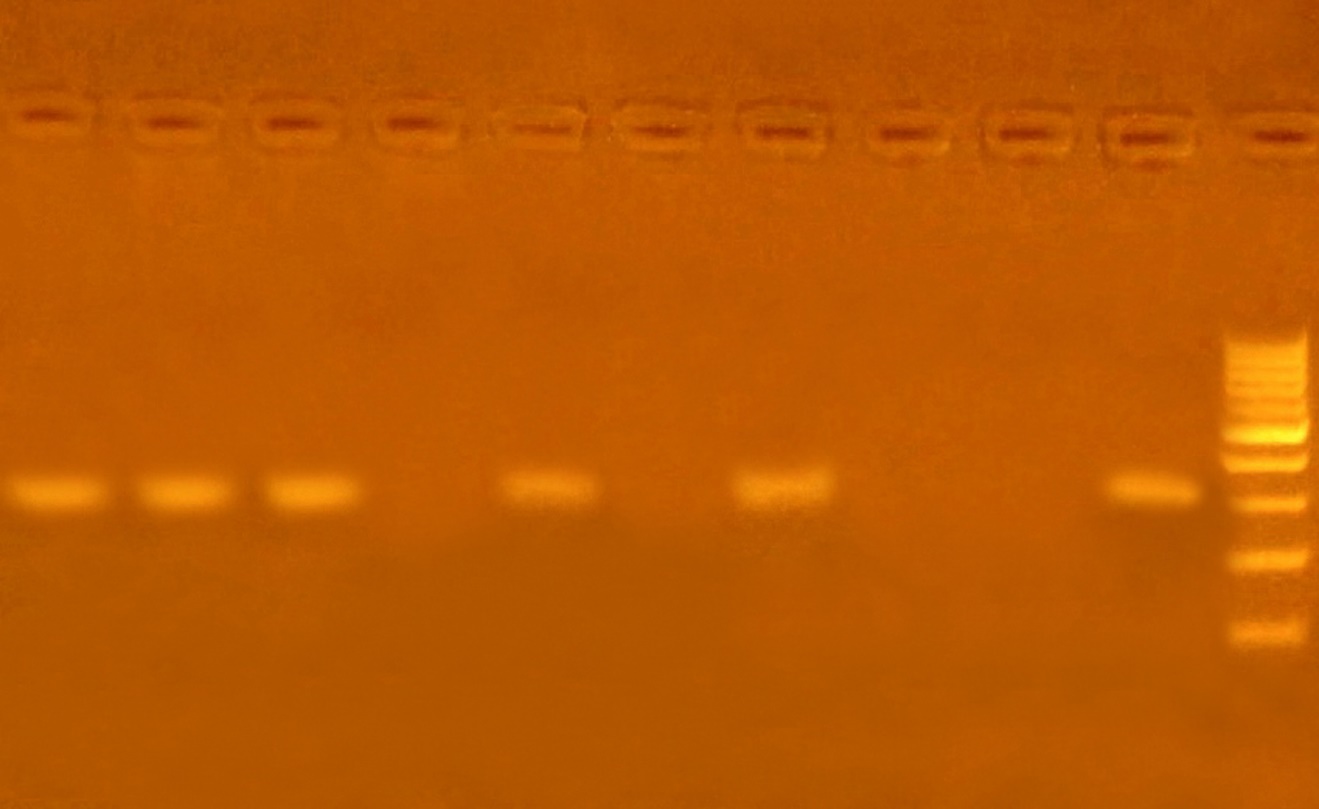


**Figure 3:** An agarose (1.5%) gel shows the 340 bp *Babesia* PCR products generated from positive samples. The positive sample is represented by lanes (2, 4, 6, 7, 8), the negative sample by lanes (1, 3, 5), the DNA size marker by lane (L), and the positive and negative controls for *Babesia* are represented by lanes (P) and (N), respectively.

**
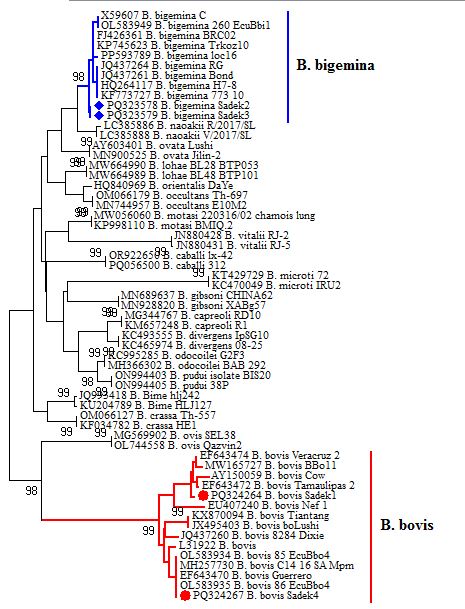
**

**Figure 4,a:** The phylogenetic tree built using the 18S rRNA gene sequences showed several clades that represent various genetic lineages of *Babesia bovis* and *Babesia bigemina*. The bar in the illustration represents the evolutionary distance divergence, which is 0.02 substitutions per site.


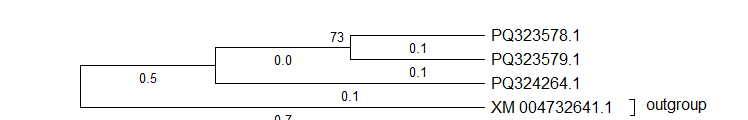


**Figure 4b:** Rooted Maximum Likelihood tree of the study isolates using *Theileria annulata* (XM_004732641.1) as an outgroup; PQ323578.1 and PQ323579.1 refer to our *Babesia bigemina* study isolates (Sadek2 and Sadek3, respectively), PQ324264.1 refers to our *Babesia bovis* study isolate (Sadek1). XM_004732641.1 represents the *Theileria annulata* sequence


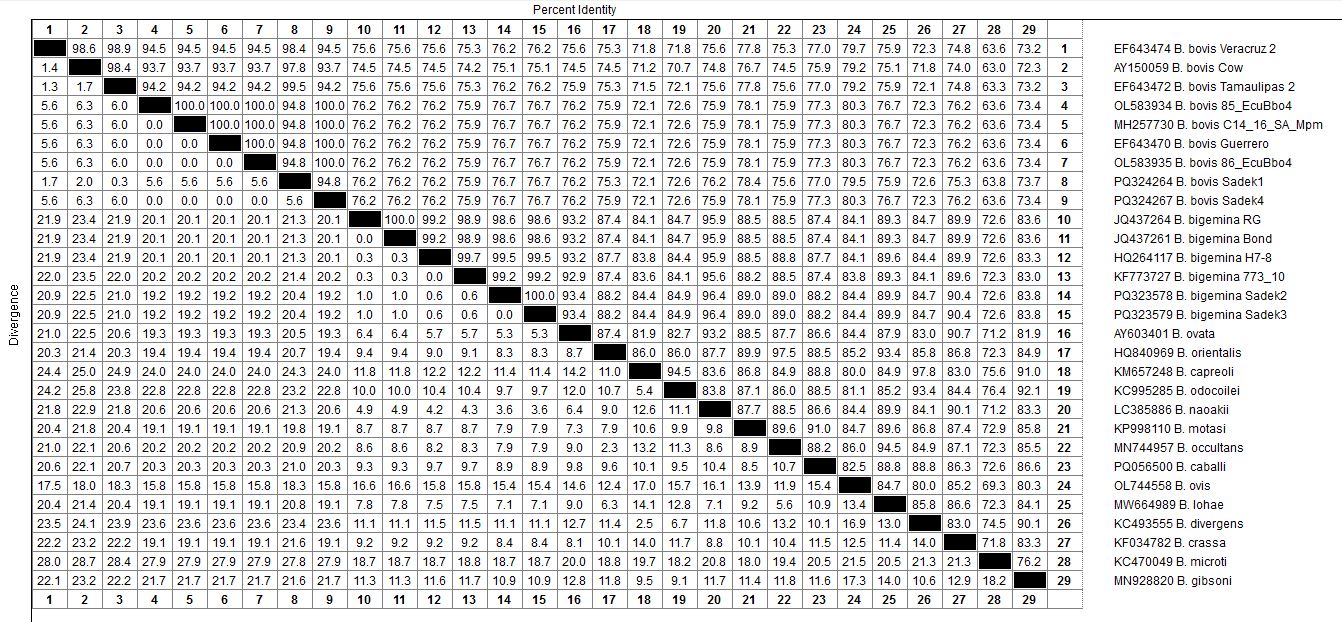


**Figure 5:** The 18S rRNA gene determines the percentages of identity for the investigated isolates of *B. bigemina* and *B. ovis* in cattle compared to other isolates worldwide.


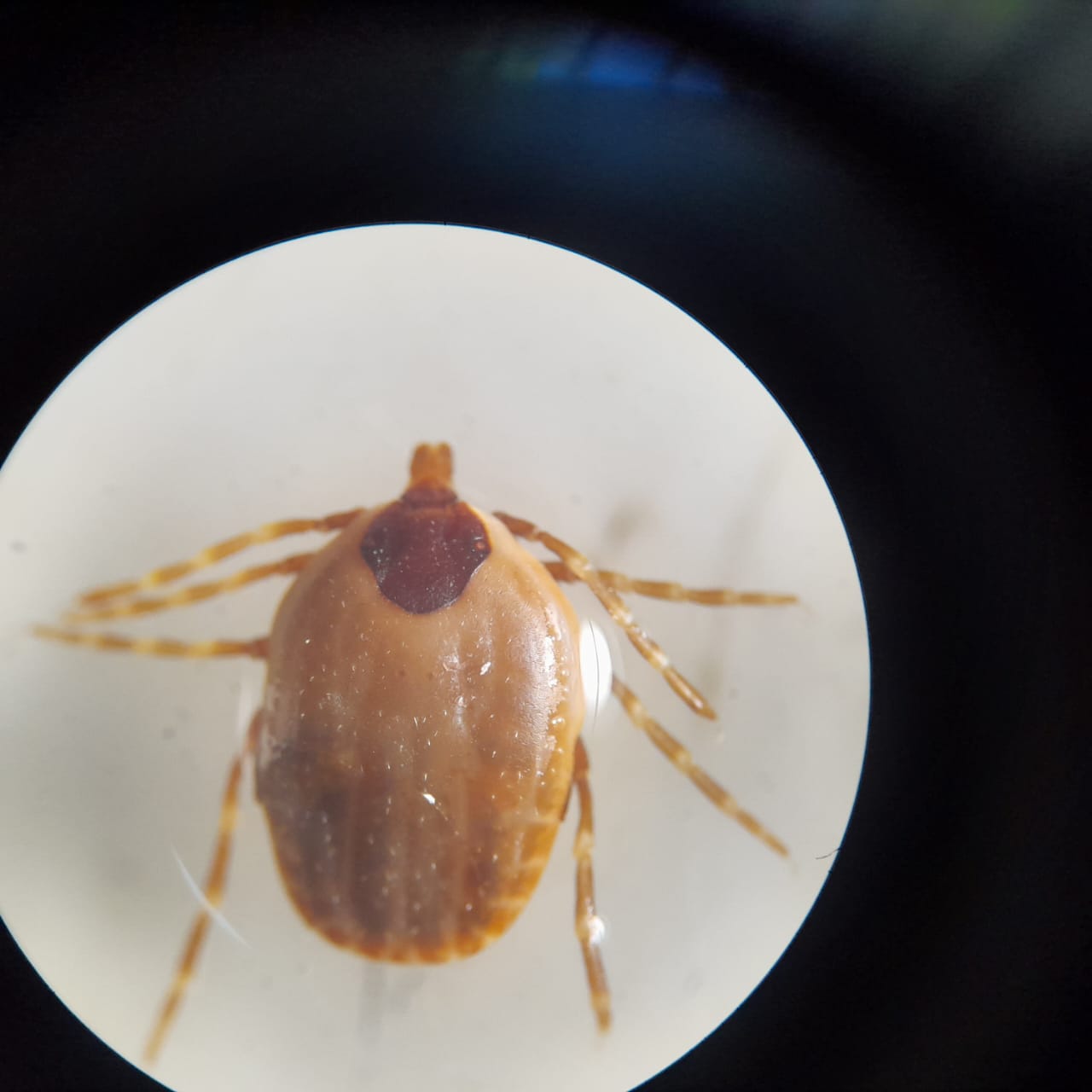


**Fig.6, a; Adult female *Hyalomma* spp*.* hard ticks; dorsal view.**

**
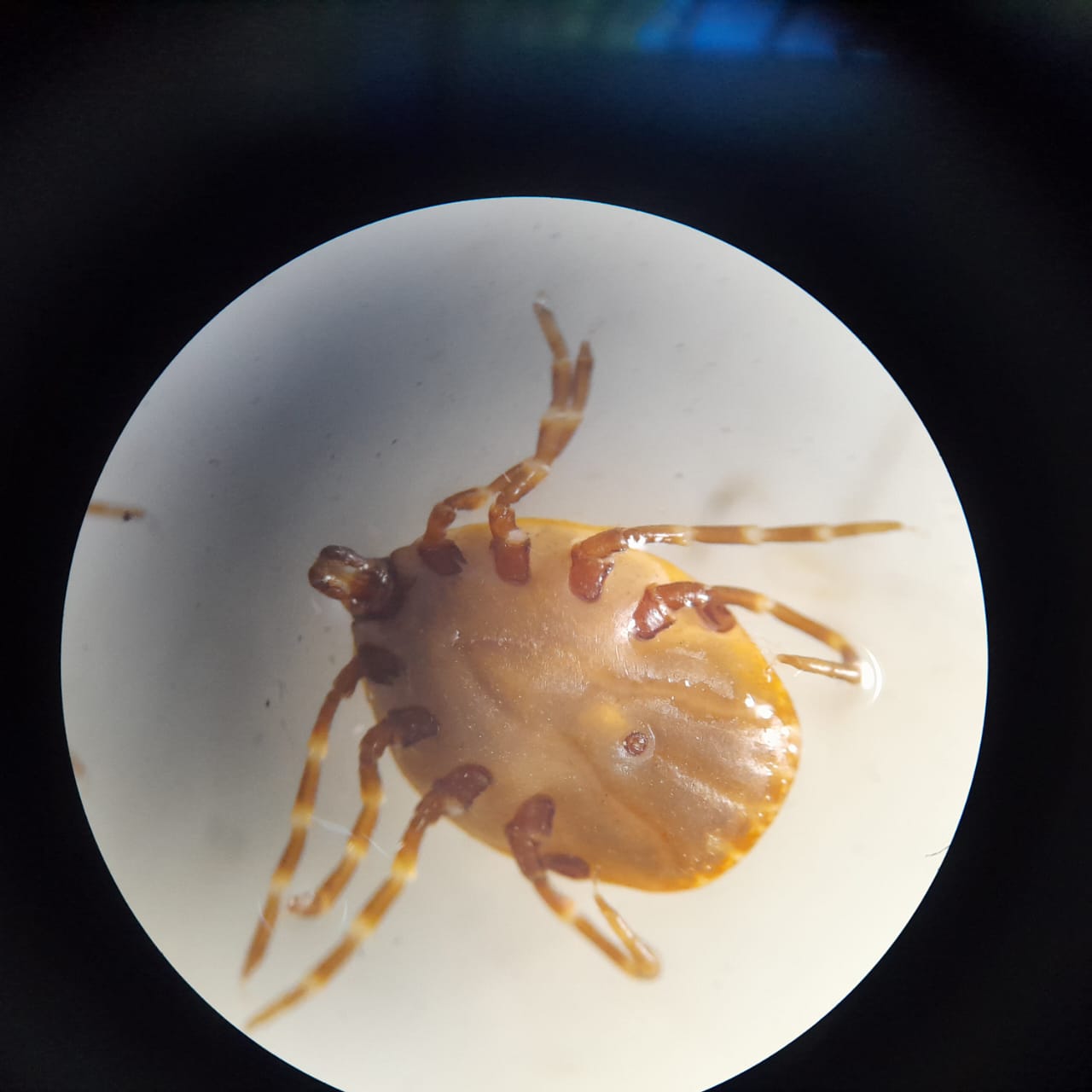
**

**Fig.6,b ; Adult female *Hyalomma* spp*.* hard ticks, ventral view.**


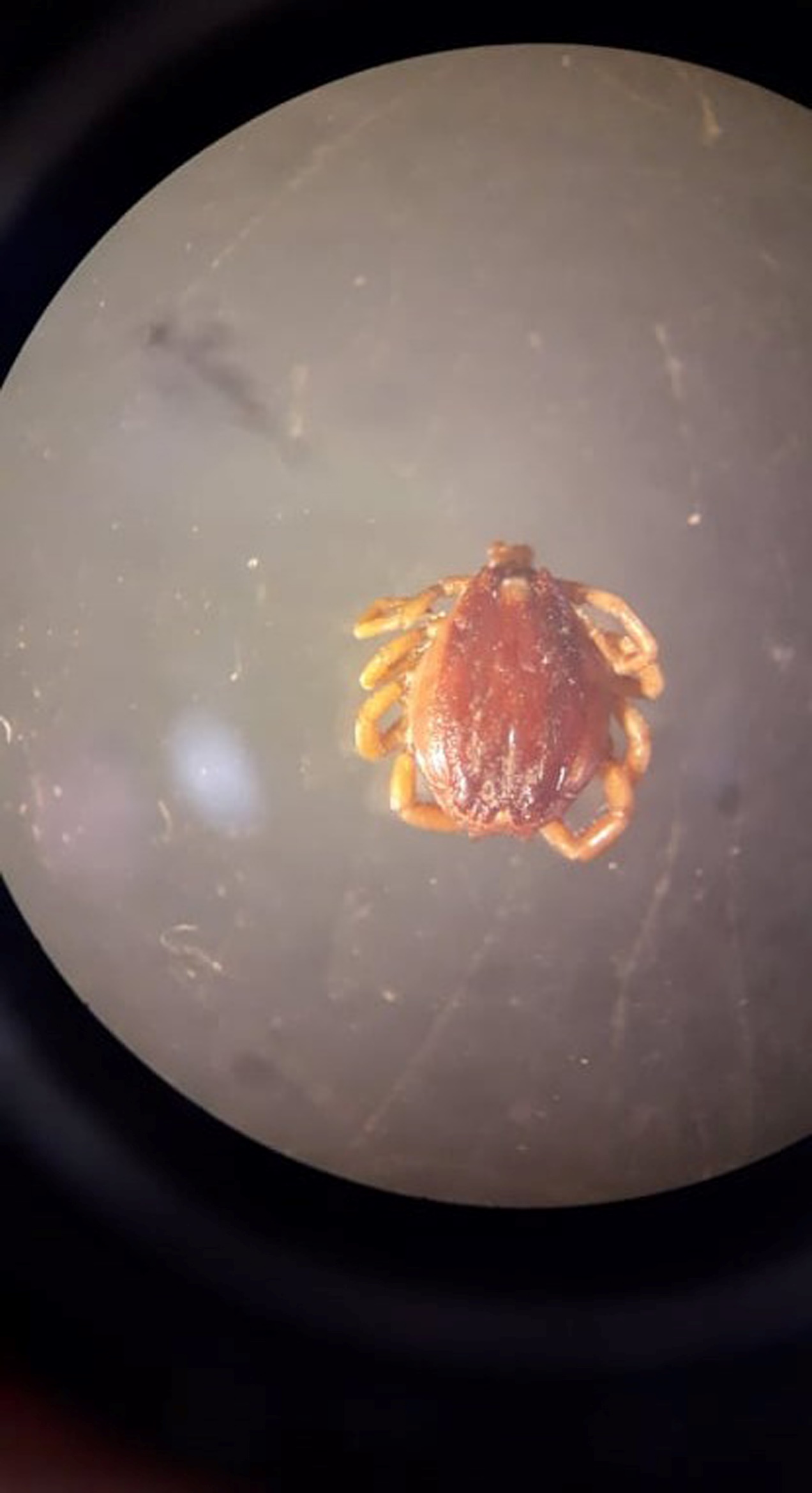


**Fig.7, a; Adult male *Hyalomma* spp*.* hard ticks, dorsal view, scale bar =2mm.**


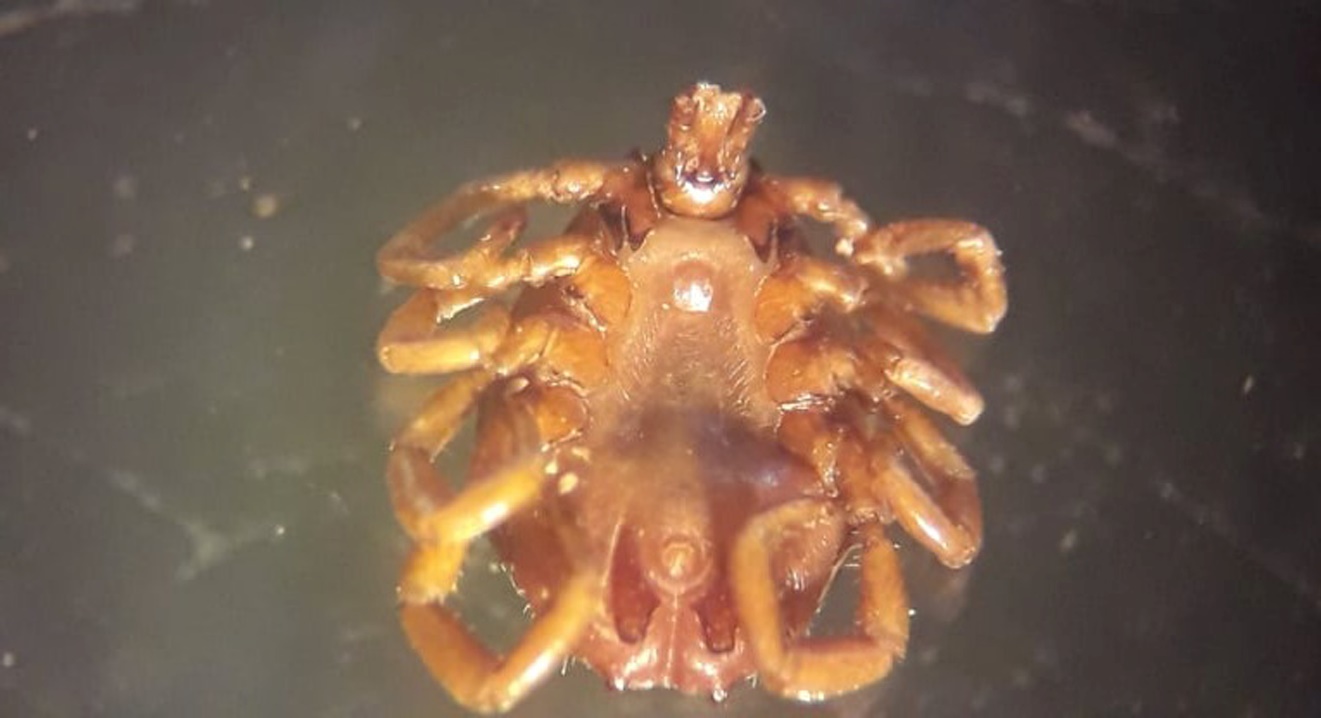


**Fig.7, b; Adult male *Hyalomma* spp*.* hard ticks, ventral view; scale bar =2mm.**
